# Fiber intake and fiber intervention in depression and anxiety: a systematic review and meta-analysis of observational studies and randomized controlled trials

**DOI:** 10.1093/nutrit/nuad143

**Published:** 2023-11-25

**Authors:** Hajara Aslam, Mojtaba Lotfaliany, Daniel So, Kirsten Berding, Michael Berk, Tetyana Rocks, Meghan Hockey, Felice N Jacka, Wolfgang Marx, John F Cryan, Heidi M Staudacher

**Affiliations:** The Institute for Mental and Physical Health and Clinical Translation (IMPACT), Food & Mood Centre, School of Medicine and Barwon Health, Deakin University, Geelong, Victoria, Australia; IMPACT, School of Medicine and Barwon Health, Deakin University, Geelong, Victoria, Australia; Department of Gastroenterology, Central Clinical School, Monash University, Melbourne, Victoria, Australia; APC Microbiome Ireland, University College Cork, Cork, Ireland; The Institute for Mental and Physical Health and Clinical Translation (IMPACT), Food & Mood Centre, School of Medicine and Barwon Health, Deakin University, Geelong, Victoria, Australia; School of Medicine, Deakin University, Geelong, Victoria, Australia; Orygen, The National Centre of Excellence in Youth Mental Health, Parkville, Victoria, Australia; Centre for Youth Mental Health, Florey Institute for Neuroscience and Mental Health, The University of Melbourne, Parkville, Victoria, Australia; Department of Psychiatry, The University of Melbourne, Victoria, Australia; The Institute for Mental and Physical Health and Clinical Translation (IMPACT), Food & Mood Centre, School of Medicine and Barwon Health, Deakin University, Geelong, Victoria, Australia; The Institute for Mental and Physical Health and Clinical Translation (IMPACT), Food & Mood Centre, School of Medicine and Barwon Health, Deakin University, Geelong, Victoria, Australia; The Institute for Mental and Physical Health and Clinical Translation (IMPACT), Food & Mood Centre, School of Medicine and Barwon Health, Deakin University, Geelong, Victoria, Australia; Centre for Adolescent Health, Murdoch Children’s Research Institute, Melbourne, Victoria, Australia; College of Public Health, Medical & Veterinary Sciences, James Cook University, Townsville, Queensland, Australia; The Institute for Mental and Physical Health and Clinical Translation (IMPACT), Food & Mood Centre, School of Medicine and Barwon Health, Deakin University, Geelong, Victoria, Australia; School of Medicine, Deakin University, Geelong, Victoria, Australia; Department of Anatomy and Neuroscience, University College Cork, Cork, Ireland; The Institute for Mental and Physical Health and Clinical Translation (IMPACT), Food & Mood Centre, School of Medicine and Barwon Health, Deakin University, Geelong, Victoria, Australia

**Keywords:** anxiety, depression, dietary fiber, gut microbiota, prebiotics

## Abstract

**Context:**

Dietary fibers hold potential to influence depressive and anxiety outcomes by modulating the microbiota–gut–brain axis, which is increasingly recognized as an underlying factor in mental health maintenance.

**Objective:**

Evidence for the effects of fibers on depressive and anxiety outcomes remains unclear. To this end, a systematic literature review and a meta-analysis were conducted that included observational studies and randomized controlled trials (RCTs).

**Data sources:**

The PubMed, Embase, CENTRAL, CINAHL, and PsychINFO databases were searched for eligible studies.

**Data extraction:**

Study screening and risk-of-bias assessment were conducted by 2 independent reviewers.

**Data analysis:**

Meta-analyses via random effects models were performed to examine the (1) association between fiber intake and depressive and anxiety outcomes in observational studies, and (2) effect of fiber intervention on depressive and anxiety outcomes compared with placebo in RCTs. A total of 181 405 participants were included in 23 observational studies. In cross-sectional studies, an inverse association was observed between fiber intake and depressive (Cohen’s d effect size [d]: −0.11; 95% confidence interval [CI]: −0.16, −0.05) and anxiety (d = −0.25; 95%CI, −0.38, −0.12) outcomes. In longitudinal studies, there was an inverse association between fiber intake and depressive outcomes (d = −0.07; 95%CI, −0.11, −0.04). In total, 740 participants were included in 10 RCTs, all of whom used fiber supplements. Of note, only 1 RCT included individuals with a clinical diagnosis of depression. No difference was found between fiber supplementation and placebo for depressive (d = −0.47; 95%CI, −1.26, 0.31) or anxiety (d = −0.30; 95%CI, −0.67, 0.07) outcomes.

**Conclusion:**

Although observational data suggest a potential benefit for higher fiber intake for depressive and anxiety outcomes, evidence from current RCTs does not support fiber supplementation for improving depressive or anxiety outcomes. More research, including RCTs in clinical populations and using a broad range of fibers, is needed.

**Systematic Review Registration:**

PROSPERO registration no. CRD42021274898.

## INTRODUCTION

Unfolding evidences suggest a potential role for dietary fibers in mood disorders,^1^^,^^2^ in addition to the established physiological health benefits of fiber, such as augmenting satiety, enhancing laxation, and regulating blood cholesterol, glucose, and insulin levels.^3^ Fibers are a heterogeneous group of compounds that comprise nondigestible plant cell wall constituents. They include cellulose, hemicelluloses, pectic substances, and also the intrinsic storage oligosaccharides, such as fructans.^4^^,^^5^ The heterogeneity of dietary fibers has led to their classification based on molecule size, chemical structure, solubility, fermentability, and viscosity.^4^^,^^5^ Because of the lack of fiber-digesting enzymes in humans, fibers escape digestion and consequently become available for microbial fermentation in the colon, which yields bioactive molecules such as short-chain fatty acids (SCFAs) as byproducts.^6^ The fermentability of dietary fibers ranges from minimally fermentable (eg, cellulose, lignin) to highly fermentable fibers (eg, fructans, pectin).^7^ Fermentable fibers that are selectively used by host microorganisms to confer health benefits are classed as prebiotic fibers (eg, fructans, galacto-oligosaccharides [GOS]).^8^

The microbiota–gut–brain axis is ever more implicated in the pathophysiology of depression and anxiety.^2^^,^^9^ The resident microbes in the gut communicate with the brain through various pathways. These include the production of signaling molecules that can modulate the immune system (eg, cytokines, SCFAs), thereby making the immune system a central intermediary between the gut microbiota and brain.^1^^,^^10^ People with psychiatric disorders often have a perturbed gut microbiota composition characterized by lower abundance of anti-inflammatory bacteria, greater abundance of pro-inflammatory bacteria, and heightened level of systemic inflammation.^11–13^ Fermentable fibers, particularly those with prebiotic properties (eg, fructans, GOS), increase the abundance of *Bifidobacterium* and *Lactobacillus,*^14^^,^^15^ taxa that have putative antidepressant and anxiolytic properties,^16^ compared with controls. These taxa also produce neurotransmitters, neuropeptides, and neuromodulators (eg, γ-aminobutyric acid, serotonin, brain- derived neurotrophic factor) that can influence depression and anxiety.^17^^,^^18^ Furthermore, higher overall fiber intake also seems to attenuate inflammation in other chronic conditions, which is reflected by reduced, systemic, pro-inflammatory (eg, C-reactive protein), and increased anti-inflammatory markers (eg, interleukin-10).^19^^,^^20^ This may be attributed to the SCFAs produced through the breakdown of fermentable fibers by the microbiota, which possess immunomodulatory and anti-inflammatory properties.^21^ Therefore, fibers, particularly more fermentable types, may have specific potential for targeting the underlying pathophysiology in depression and anxiety.

The potential role of dietary fiber on depression and anxiety has been assessed in both observational studies and randomized controlled trials (RCTs). Findings from observational studies have shown mixed results for the association between higher total dietary fiber intake, including different types or sources of fiber (eg, soluble and insoluble or fruit and vegetable fiber), and depression and/or anxiety.^22–26^ A meta-analysis of these observational studies showed that greater fiber intake was associated with lower odds for depressive outcomes^27^; however, owing to the methodological issues in search strategy, data extraction, and data analysis, an editorial pointed out the need for additional studies.^28^ Conversely, the number of RCTs conducted to date have been limited and have primarily evaluated the effects of fermentable fibers, yielding inconsistent results.^29–31^ Indeed, a previous meta-analysis examining only prebiotic fibers for effect on depression and anxiety did not show superiority of their supplementation over placebo.^32^ RCT data on fibers overall, or of other types of fermentable fibers, are yet to be synthesized.

In addition to the limitations of extant data on this topic, there is a dearth of robust summary evidence for the role of dietary fiber on depressive and anxiety outcomes covering both observational studies and RCTs. Importantly, given the growing recognition of the role of diet in the prevention and management of mood disorders, it is imperative to have a better understanding of how key dietary components, such as fiber, may influence depressive and anxiety outcomes. Therefore, we conducted a systematic literature review and meta-analysis of both observational studies and RCTs.

## METHODS

### Literature search

A broad systematic literature search was conducted using a variety of terms, subject headings, and synonyms relevant to the key concepts of this manuscript (fiber and clinical entities of anxiety and depression). The full search string is provided in Appendix S1 in the Supporting Information online. The electronic databases PubMed, Embase, the Cochrane Central Register of Controlled Trials (CENTRAL), CINAHL (via EBSCOhost), and PsychINFO were searched. Furthermore, hand-searching of relevant published articles was performed to identify additional studies. Only articles published in English were included. Articles from conference proceedings, thesis, reports, unpublished grey literature, and reviews were excluded. The study was preregistered in PROSPERO (no. CRD42021274898).

### Study selection

Observational studies and RCTs were considered. Studies of healthy and clinical populations of adults aged ≥18 years in whom fiber intake and depressive or anxiety outcomes were measured were included (Table 1).

**Table 1:** PICOS criteria for inclusion and exclusion of observational studies and randomized controlled trials

	Criteria	
Parameter	Observational studies	Randomized controlled trials
Population	Human participants, both healthy and diseased (eg, individuals with type 2 diabetes mellitus)	Human participant, both healthy and diseased (eg, individuals with type 2 diabetes mellitus)
Intervention	Dietary fiber intake	Whole-diet fiber interventions or fiber supplements
Comparator	Low dietary fiber intake	Habitual or sham diet, low-fiber control diet, or placebo (eg, maltodextrin)
Outcomes	Depressive and anxiety outcomes	Depressive and anxiety outcomes

#### Observational study selection

Cross-sectional, longitudinal, case-control, and retrospective studies were deemed eligible. Studies assessing dietary fiber intake using a comprehensive dietary assessment method (eg, food frequency questionnaires [FFQs] or 24-h recall) and depressive and/or anxiety outcomes by clinical diagnosis or self-reported validated questionnaires (eg, Patient Health Questionnaire, Depression Anxiety Stress Scale, Hospital Anxiety Depression Scale) were deemed eligible, as were studies that included the use of antidepressive or anxiolytic drugs along with a validated questionnaire.

#### Randomized controlled trial selection

Crossover and parallel design and quasi-RCTs were deemed eligible. RCTs assessing fiber interventions attained through either whole-diet modification (ie, delivered through dietary counselling, provision of high-fiber food, or controlled feeding) or fiber supplements (ie, natural, synthetic, isolated, or fiber mixes) and depressive and/or anxiety outcomes by a validated method (eg, self-reported questionnaire, clinician diagnosis) were included. RCTs assessing the effects of synbiotics (ie, combination of prebiotics and probiotics) were excluded. RCTs assessing increased fiber intake combined with other interventions, such as energy restriction or exercise, were excluded unless the RCT also included a fiber and control arm.

### Screening and data extraction

Search results were managed in the reference management software Endnote and the web application Covidence. Studies retrieved using the search strategy were screened against study inclusion and exclusion criteria by 3 reviewers (H.A., M.H., T.R.). Full text of these potentially eligible studies was independently assessed for eligibility by 2 review team members (H.A., D.S.), and disagreement over the eligibility of studies was resolved through discussion with a third reviewer (H.M.S., W.M., or M.B.). Data for observational studies and RCTs were extracted by 2 independent authors (H.A., K.B.) using 2 stand-alone, prepiloted data extraction forms. General information such as author name, journal, year published, and study design were captured by both data extraction forms.

In addition, the data extraction form for observational studies collected the following: (1) dietary fiber intake assessment methods, (2) depression and anxiety assessment methods, and (3) statistical estimates, which included odds ratios, hazard ratios (HRs), β-coefficient, or means and their corresponding standard errors (SEs), standard deviations (SDs), or 95% confidence intervals (CIs), considered from the statistical model with maximum covariate adjustments. Several factors were considered during data extraction. When dietary fiber intake was treated both as categorical and continuous, statistical estimates from the model that treated fiber as a continuous variable were extracted^33^ for statistical robustness.^34^ When fiber was categorized (eg, tertiles, quartiles), the corresponding statistical estimates for the highest category compared with the lowest category were extracted.^24^^,^^25^^,^^35–42^ When a study used different methods to assess the outcome, statistical estimates for the models using the most robust method (eg, clinician-diagnosed depression was chosen over self-reported) were extracted.^35^^,^^36^^,^^42^ When studies reported statistical estimates by population subsets (eg, menopause status, sex),^33^^,^^40^^,^^43^^,^^44^ those data were extracted separately; however, if the study also reported estimates for the total population, the statistical estimates for the total population were extracted.^23^^,^^38^^,^^45^

The data extraction form for RCTs was used to collect the following information: (1) intervention type (ie, whole dietary fiber intervention or supplementation); (2) the specific type of fiber evaluated in supplementation trials (eg, fructans, GOS, polydextrose); (3) fiber content (ie, total dietary fiber intake or estimated fiber content for whole-fiber intervention trials or supplement dose for fiber supplementation trials); (4) outcome assessment methods; and (5) statistical estimates, which included before and after intervention means and their corresponding SEs, or 95%CIs for both intervention and control groups to compare the change in outcomes between groups. When a study did not report statistical estimates in the text, the required estimates were calculated from figures presented^29^; where data were missing, the corresponding author was contacted.^46^

### Risk-of-bias assessment

The National Institute of Health Quality Assessment of Observational Cohort and Cross-Sectional Studies tool was used to assess risk of bias for cross-sectional and longitudinal studies. This tool entails 14 questions concerning the internal validity of the study.^47^ Factors such as study design, confounders, and follow-up duration were considered during critical appraisal, and the study quality was rated as either “good,” “fair,” or “poor.” The National Institute of Health Quality Assessment of Case-Control Studies tool was used to assess risk of bias in case-control studies.^47^ This tool entails 12 questions and captures information that is central in assessing study quality, which, likewise, was rated as “good,” “fair,” or “poor.”

Version 2 of the Cochrane Collaboration Risk of Bias tool was used to assess risk of bias of RCTs.^48^ This tool entails 5 domains with signalling questions that inform the risk of bias from randomization, deviations of intended interventions, missing outcome data, and measurement of outcome and bias in reporting results, using a scoring algorithm. The risk of bias in each domain was classed as “high risk,” “low risk,” or “some concerns.” A study was judged as low risk of bias when all 5 domains had low risk of bias, whereas a study was judged as high risk of bias when at least 1 of the 5 domains had high risk of bias or multiple domains with some concerns. Conflicting judgment about studies was resolved collaboratively.

### Statistical analysis

#### Preprocessing data for meta-analysis

The different statistical estimates reported by studies are detailed in Table S1 in the Supporting Information online. These estimates were converted to Cohen’s *d* effect sizes (ESs) and their corresponding SE using the *esc* package in R statistical software (version 4.01; R Foundation for Statistical Computing) prior to meta-analysing.^49^

For observational studies, when a study reported results separately based on sex,^40^^,^^43^^,^^44^ and menopause (ie, early perimenopause and premenopause),^33^ the ESs and SEs for subsets were calculated separately and combined using fixed effect meta-analysis. When studies reported HRs,^24^ the prevalence ratio for the condition (eg, depression) was calculated and if this value was less than 10%, the HR was considered equal to the OR and subsequently converted to ES and SE.^50^

For RCTs, when 2 different types of fiber supplementation (ie, fructo-oligosaccharide [FOS] and GOS) were tested in a multiarm RCT,^51^ the ES and inflated SE for each supplement arm were calculated and subsequently pooled^52^; however, for subgroup analysis, each type of fiber supplement arm was treated separately. When 2 doses of a fiber supplement were tested in a multiarm RCT, the statistical estimates for the highest dose was used for ES and SE calculation.^53^

#### Meta-analysis

For each outcome of interest (ie, depression and anxiety), we conducted separate meta-analyses by study design (cross-sectional, longitudinal, case-control, and RCTs), using a random-effects model to account for heterogeneity between studies. Heterogeneity was assessed using the *I^2^* statistic, and an *I*^2^ value greater than 75% was considered substantial heterogeneity. Forest plots were used to visualize the ESs and CIs of the included studies, along with the summary ES.

Subgroup analyses were conducted to examine the impact of (1) risk of bias and, in fiber-supplementation RCTs only: (2) fiber supplementation dose (low: ≤5.5 g/d vs high: >5.5 g/d), and (3) the fiber types evaluated.

Publication bias was assessed using funnel plots and Egger’s regression test. Funnel plots were generated only for meta-analyses that included more than 10 studies.^14^^,^^54^ Outliers were identified through funnel plots, and sensitivity analysis was conducted by removing outlier studies to identify whether the overall outcome was driven by a particular study. In addition, sensitivity analyses were conducted on cross-sectional studies by removing studies with populations with disease or older populations to identify if these factors affected the overall outcome.

## RESULTS

**Figure 1:**
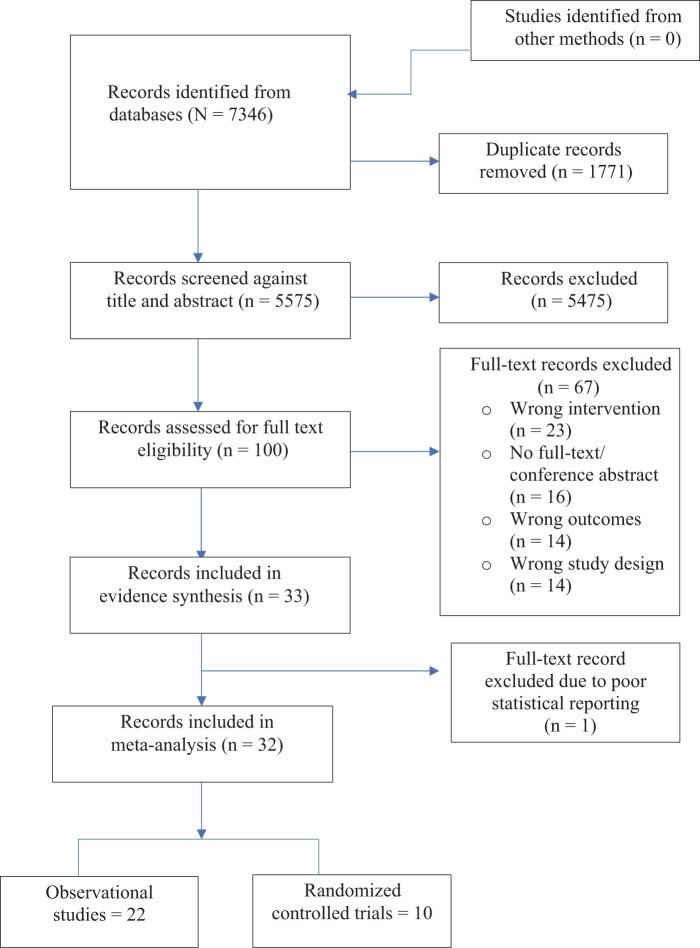
Preferred Reporting Items for Systematic Reviews and Meta-Analyses flow diagram.

The search strategy resulted in 5575 deduplicated articles, which were screened against the predefined eligibility criteria. In total, 33 eligible studies were identified (23 observational studies and 10 RCTs). All 33 studies were used in evidence synthesis; however, of the 33 studies, only 32 were included in meta-analysis, because 1 study^55^ did not contain the data required for meta-analysis (Figure 1).

### Observational studies

#### Study characteristics

A total of 181 405 participants were included in the 23 studies (Table 2).^22–26^^,^^33^^,^^35–45^^,^^55–60^ Of the 23 studies, 16 were cross-sectional,^22^^,^^23^^,^^25^^,^^26^^,^^33^^,^^35–40^^,^^43–45^^,^^56^^,^^57^ 3 were longitudinal^24^^,^^41^^,^^42^ with follow-up periods ranging between 3 to 17 years, and 4 were case-control studies.^55^^,^^58–60^ All studies assessed total dietary fiber intake. Some studies assessed a particular fiber type (eg, soluble and insoluble, prebiotic) or fiber source (eg, fruits, vegetable)^22^^,^^24–26^^,^^35–37^^,^^40^^,^^42^ in addition to total fiber intake. In this review, we only considered total fiber and findings about different fiber types, and depressive and/or anxiety outcomes are summarized and reported separately from our main findings (Table S2 in the Supporting Information online). In total, 20 studies reported depressive outcomes^23–26^^,^^33^^,^^35–37^^,^^39–43^^,^^45^^,^^55–60^ and 3 studies reported both depressive and anxiety outcomes.^22^^,^^38^^,^^44^ Most studies had a healthy adult population at baseline, whereas 1 study was conducted with individuals diagnosed with an alcohol-use disorder^22^ and 1 with individuals diagnosed with HIV.^56^ Six studies used data only from a female population for analysis.^26^^,^^33^^,^^41^^,^^42^^,^^45^^,^^60^ Of these, 2 studies included postmenopausal women.^41^^,^^42^ Five studies included an elderly population (age range, 70–75 years) for analysis.^35^^,^^39^^,^^43^^,^^58^^,^^59^

**Table 2:** Study characteristics: observational studies

Reference	Country	Population, sample size	Mean age (y)	Female sex (%)	Exposure	Exposure assessment method	Outcome	Outcome assessment method	Adjustments	Model
Cross-sectional studies										
Amadieu et al (2021)^22^	Belgium	Alcohol use disorder individuals (n = 48)	47	37.5	Total dietary fiber	Three 24-h recalls	Depression and anxiety	Depression: Beck Depression Inventory Anxiety: State-Trait Anxiety Inventory	Age, sex, educational level, energy intake, BMI, tobacco, and alcohol consumption	Linear regression
Chrzastek et al (2020)^43^	Poland	Older adults (n = 813)	75	73	Total dietary fiber	24-h recall	Symptoms of depression	15-item GDS (GDS score > 5)^a^	Age, education years, waist circumference, Cognitive function, and chronic obstructive pulmonary disease	Logistic regression (analyses for women and men performed separately)
Eissenstat et al (2020)^23^	United States	Healthy adults (n = 4747)	≥18	53	Total dietary fiber	Two 24-h recalls	Symptoms of depression	PHQ-9	Sex, age, education, income, birthplace, and race	Linear regression
Fang et al (2013)^26^	United States	Healthy adults (n = 225)	27	100	Total dietary fiber	Three 24-h recalls	Symptoms of depression	20-item CESD	Prior condition, age, race, education, marital status, hormonal contraceptive use, income, and history of heart disease	Linear regression
Gopinath et al (2016)^35^	Australia	Older adults (n = 1504)	73	62	Total dietary fiber	145-item, self-administered FFQ	Symptoms of depression	10-scale CESD or use of antidepressants (score ≥ 10)^a^	Age, sex, energy, cognitive impairment, walking disability, receiving pension, and antidepressant use, previous history of stroke and arthritis	Logistic regression
Kim et al (2020)^36^	Korea	Healthy adults (n = 546)	42	65	Total dietary fiber	FFQ	Clinical depression	Diagnosis of depression by a physician	Age, sex, economic status, education, smoking status, alcohol consumption, physical activity, subjective health status, BMI, and total energy intake	Logistic regression
Kim et al (2021)^45^	Korea	Healthy adults/premenopausal women (n= 5807)	47	100	Total dietary fiber	24-h recall	Depression	PHQ-9 (score ≥ 10)^a^	Age, BMI, education level, marital status, household income, smoking status, alcohol use, adequate physical activity, and chronic disease status	Logistic regression
Li et al (2020)^33^	United States	Healthy adults/premenopausal women (n = 3054)	46	100	Total dietary fiber	FFQ	Symptoms of depression	20-item CESD	Age, race/ethnicity, total family income, education, sport, BMI, dietary total caloric intake, use of antidepressant, SHBG, and FSH	Linear regression (analyses for premenopausal and early perimenopausal women performed separately)
Miki et al (2016)^37^	Japan	Healthy adults (n = 1977)	42	11	Total dietary fiber	Brief self-administered diet history questionnaire	Symptoms of depression	Japanese version of CESD (score ≥16)^a^	Age, sex, site, marital status, job grade, and other factors^b^	Logistic regression
Purnomo et al (2021)^56^	Australia	HIV individuals (depressed vs nondepressed) (n = 58)	43	3.4	Total dietary fiber	FFQ	Symptoms of depression	10-item CESD (score ≥ 10)^a^	–	Mann-Whitney *U* test
Rintamaki et al (2014)^44^	Finland	Healthy vs individuals with depressive and anxiety disorder (n = 5351)	48	59	Total dietary fiber	128- item FFQ	Depression and anxiety	A structured, computer-aided mental health interview	Age, education, social support smoking, and energy intake	General linear model (analyses for men and women performed separately)
Saghafian et al (2021)^38^	Iran	Healthy adults (n = 3363)	36	58	Total dietary fiber	106-item FFQ	Symptoms of depression and anxiety	Iranian version of HADS (score > 21)^a^	Age, sex, energy intake, and other factors^c^	Logistic regression
Woo et al (2006)^39^	Hong Kong	Older adults (n = 3395)	72	44	Total dietary fiber	7-day FFQ	Depression	Face-to-face interviews, using a validated Chinese version of GDS (score ≥ 8)^a^	CSID score, age, sex, education level, socioeconomic status, and number of medical diseases	Logistic regression
Xia et al (2021)^40^	China	General adults (n = 24 306)	41	46	Total dietary fiber	100-food item modified FFQ	Symptoms of depression	Chinese version of the Zung SDS (score >45)	Age, BMI, type 2 diabetes, hypertension, hyperlipidemia, physical activity, and other factors^d^	Logistic regression (analyses for men and women performed separately)
Xu et al (2018)^25^	United States	Civilian noninstitutionalized US population (16 807)	50	57	Total dietary fiber	24-h recall	Symptoms of depression	PHQ-9 (score ≥ 10)^a^	Age, sex, race, marital status and other factors^e^	Logistic regression
Yun et al (2021)^57^	Korea	General adults (n = 10 106)	65	58	Total dietary fiber	Diet questionnaire (un-specified)	Symptoms of depression	PHQ-9	Total food intake, sex, income, education, and marital status	Linear regression
Longitudinal studies										
Gangwisch et al (2015)^41^	Columbia	Postmenopausal women (n= 69 954)	64	100	Total dietary fiber	145-item FFQ	Depressive symptoms	Burnam 8-item scale (standard threshold of 0.06)^a^	Nutrient density, race/ethnicity, education, income, BMI, and other factors^f^	Logistic regression (3-y follow-up)
Perez-Cornago et al (2016)^24^	Spain	Healthy adults (n = 14 539)	38	59	Total prebiotic	136-item semi-quantitative FFQ	Depression	Self-reported, physician-diagnosed	Age, sex, BMI, smoking, marital status, personality traits, unemployment, and living alone	Multivariable Cox proportional hazard (9.3-y follow-up)
Ramin et al (2020)^42^	United States	Postmenopausal women (n= 14 129)	60	100	Total dietary fiber	127-food-item Harvard FFQ	Depressive symptoms	Mental Health Score	Total calorie, age, education, alcohol, physical activity, antidepressant use, and WHR	Linear regression (17-y follow-up)
Case-control studies										
Gougeon et al (2017)^58^	Canada	Older adults (n = 316; depressed vs nondepressed)	75	61	Total dietary fiber	24-h recall	Depression	GDS or use of anti-depressants (score ≥ 11)^a^	Physical activity, functional autonomy, and stressful life events	General linear model
Guligowska et al (2016)^59^	Poland	Older adults (n = 130; depressed vs nondepressed)	71	76	Total dietary fiber	24-h recall	Depression	15-item GDS (cutoff for depression not reported)^a^	NA	Mann-Whitney *U* test
Othman et al (2018)^55^	Tunisia	Adults (n = 100; depressed vs nondepressed)	44.5	67	Total dietary fiber	Feeding (diet) history	Depression	HADS and PHQ-9 (cutoff for depression not reported)^a^	NA	Student’s *t* test
Park et al (2010)^60^	South Korea	College students (n = 130; depressed vs nondepressed)	20	100	Total dietary fiber	3-d recall	Depression	Korean version of CESD (score >16)^a^	NA	Student’s *t* test

a The cutoff scores for establishing either depressive symptoms or depression.

^b,c,d,e,f^denotes the covariates used in statistical model adjustments in addition the ones listed in the table (further elaborated in Appendix S1).

*Abbreviations: BMI, body mass index; CESD, Centre for Epidemiologic Studies Depression; CSID, Community Screening Instrument for Dementia; FFQ, food frequency questionnaire; FSH, follicle-stimulating hormone; GDS, Geriatric Depression Scale; HADS, Hospital Anxiety and Depression Scale; NA, not applicable; PHQ, Patient Health Questionnaire; SDS, Self-Rating Depression Scale; SHBG, sex hormone binding globulin; WHR, waist to hip ratio.*

Five methods were used to assess total fiber intake, of which FFQs were the most used method (n = 11 of 23). A total of 12 methods were used to assess depressive and anxiety outcomes. Self-reported questionnaires were the most used method (n = 19 of 23) (Figure S1 in the Supporting Information online). Risk of bias of observational studies varied across studies (Table S3 in the Supporting Information online). Of the studies rated as good^22^^,^^24^^,^^25^^,^^33^^,^^35^^,^^37^^,^^38^^,^^40^^,^^43^^,^^58^ and as fair,^23^^,^^26^^,^^36^^,^^39^^,^^41^^,^^42^^,^^44^^,^^45^^,^^59^ the majority defined their research question, study population, exposure, and outcome variable clearly, included an eligible participant rate greater than 50% and adjusted for potential confounders in the analysis. Four studies were rated as poor^55–57^^,^^60^; these studies either did not clearly define the study population or exposure variables, or the authors did not adjust for confounding in their analysis.

#### The association between total dietary fiber intake and depressive and anxiety outcomes

**Figure 2:**
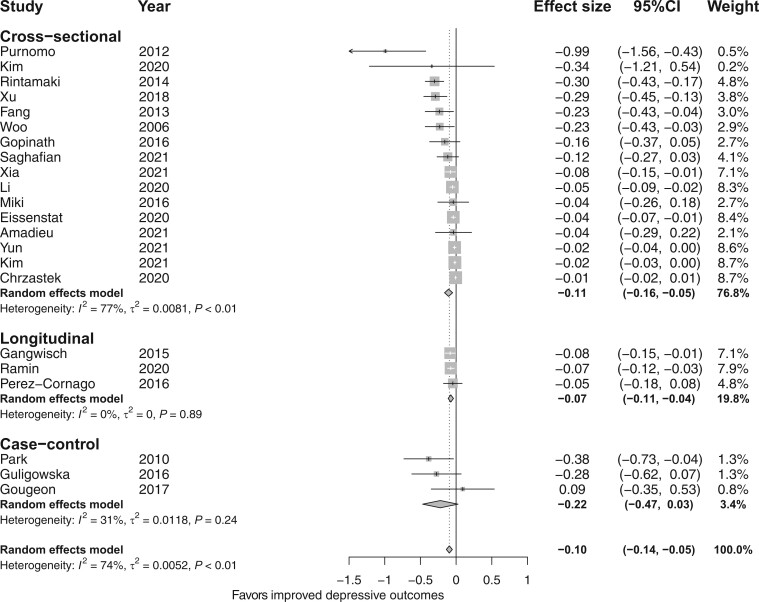
**Forest plot of observational studies investigating the association between fiber intake and depressive outcomes by study design**. Box size represents study weight and diamonds represent overall effect sizes and 95%CIs.

**Figure 3:**
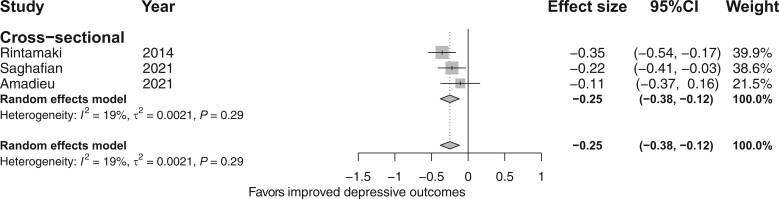
**Forest plot of cross-sectional studies investigating the association between fiber intake and anxiety outcomes**. Box size represents study weight and diamonds represent overall effect sizes and 95%CIs.

The meta-analysis of 16 cross-sectional studies (n = 82 107) found a significant inverse association between total fiber intake and depressive outcomes (*d* = −0.11; 95%CI, −0.16, −0.05; *I*^2^ = 74%; Figure 2),^22–26^^,^^33^^,^^35–45^^,^^55–60^ but the ES for this association was small. There was also a significant inverse association between total fiber intake and anxiety outcomes (*d* = −0.25; 95%CI, −0.38, −0.12; *I*^2^ = 19%; Figure 3);^22^^,^^38^^,^^44^ however, only 3 cross-sectionals studies^22^^,^^38^^,^^44^ were included in the analysis and the ES for this association was also small. The meta-analysis of 3 longitudinal studies (n = 98 622) found a significant inverse association between total fiber intake and depressive outcomes (*d* = −0.07; 95%CI, −0.11, −0.04; *I*^2^ = 0%; Figure 2); however, the ES for this association was small. The meta-analysis of 3 case-control studies (n = 676) showed no difference in total fiber intake between depressed and nondepressed or control groups (*d* = −0.22; 95%CI, −0.47, 0.03; *I*^2^ = 31%; Figure 2). None of the longitudinal and case-control studies assessed anxiety outcomes.

Subgroup analysis based on risk of bias was only possible in cross-sectional studies for depressive outcomes, because of the smaller number of studies for other observational study designs and cross-sectional studies evaluating anxiety outcomes. A significant inverse association between fiber intake and depressive outcomes was reported in the good risk-of-bias group as opposed to the poor and fair groups; however, there were no overall differences when comparing the 3 risk-of-bias groups (*x*^2^ =1.65; degrees of freedom [df] = 2; *P *=* *0.44), and there was higher heterogeneity in each subgroup compared (see Figure S2 in the Supporting Information online).

### Randomized controlled trials

#### Study characteristics

A total of 740 participants were included in 10 RCTs (Table 3).^29–31^^,^^46^^,^^51^^,^^53^^,^^61–64^ All RCTs were placebo-controlled trials, of which 6 had a parallel design^29^^,^^30^^,^^51^^,^^61–63^ and 4 were of crossover design.^31^^,^^46^^,^^53^^,^^64^ The intervention duration of RCTs ranged from 2 to 8 weeks. Four RCTs were conducted with healthy individuals,^31^^,^^46^^,^^51^^,^^62^ 3 were conducted with individuals with gastrointestinal symptoms (ie, irritable bowel syndrome [IBS] and functional gastrointestinal symptoms)^29^^,^^53^^,^^64^ and 1 each included individuals with type 2 diabetes mellitus,^61^ mild to moderate depression,^30^ and coronary artery disease.^63^ Three studies involved female participants only.^31^^,^^61^^,^^62^ The average age of the participants was 38 (range, 21–51) years.

**Table 3:** Study characteristics: clinical trials

Reference	Country	Mean age (y)	Female sex (%)	Population, sample size	Study design and duration	Intervention and dosage	Control/placebo and dose	Outcome assessment method	Outcome	Adherence
Azpiroz et al (2017)^29^	France and Spain	41.5	75	Patients with IBS (n = 79)	Parallel 4-wk	scFOS, 5 g/d	Maltodextrin, 5 g/d	HADS	Changes in HADS scores for anxiety and depression	NR
Farhangi et al (2018)^61^	Iran	49.4	100	Patients with T2DM (n = 62)	Parallel 8-wk	NUTRIOSE^®^06 resistant dextrin, 10 g/d	Maltodextrin, 10 g/d	DASS	Change in overall DASS score	NR
Ibarra et al (2016)^31^	The Netherlands	27.4	100	Healthy adults (n = 32)	Crossover, 150 min	Polydextrose, 12.5 g in yogurt	Glucose syrup in yogurt to match the calorie content in the intervention	POMS-32	Change in depression score	NR
Johnstone et al (2021)^62^	United Kingdom	21.5	100	Healthy adults (n = 48)	Parallel 4-wk	GOS, 7.5 g/d	Maltodextrin, 7.5 g/d	STAI	Change in trait anxiety	M^a^
Kazemi et al (2019)^30^	Iran	36.5	71	Patients with mild to moderate depression (n = 72)	Parallel, 8 wk	GOS, 5 g/d	Mixture of xylitol and maltodextrin, 5 g/d	BDI	Chang in BDI score	M^b^
Moludi et al (2021)^63^	Iran	51	40	Patients with CAD (n = 96)	Parallel 8-wk	Inulin, 15 g/d	Maltodextrin 15 g/d	BDI-II and STAI-Y	Change in BDI scores for depression and STAI-Y scores for anxiety	M^b^
Schmidt et al (2014)^51^	United Kingdom	24	51	Healthy adults (n = 45)	Parallel 3-wk	FOS and GOS, 5.5 g/d	Maltodextrin, 5.5 g/d	STAI-trait	Change in STAI-trait scores for anxiety	M^b^
Silk et al (2009)^53^	United Kingdom	54	64	Patients with IBS (n = 44)	Cross-over 4-wk	trans-GOS, 7 g/d	Maltodextrin, 7 g/d	HADS	Changes in HADS scores for anxiety and depression	M
Vulevic et al (2018)^64^	United Kingdom	35	57	Adults with GI symptoms (n = 120)	Cross-over 2-wk	Prebiotic, B-GOS (Bimuno) 2.75 g/d	Maltodextrin, 2.75 g/d	HADS	Change in HADS scores	M^b^
Smith et al (2005)^46^	United Kingdom	32	51	Healthy adults (n = 142)	Cross over 2-wk	Oligofructose-enriched inulin, 10 g/d	Maltodextrin, 10 g/d	HADS	Changes in HADS scores for anxiety and depression	M

a Method of compliance assessment or compliance rate not reported.

b Compliance rate >80%.

*Abbreviations: BDI-II, revised Beck Depression Inventory; CAD, coronary artery disease; DASS, Depression, Anxiety, and Stress Scale; FOS, fructo-oligosaccharide; GI, gastrointestinal; GOS, galacto-oligosaccharide; HADS, Hospital Anxiety Depression Scale; IBS, irritable bowel syndrome; M, monitored; NR, not reported; POMS-32, 32-item Profile of Mood States questionnaire; scFOS, short-chain fructo-oligosaccharides; STAI, State-Trait Anxiety Inventory; STAI-Y, State-Trait Anxiety Inventory form Y; T2DM, type 2 diabetes mellitus.*

All interventions were delivered through fiber supplementation and examined fermentable fibers. Of these, 8 trials examined prebiotic fibers: 4 evaluated GOS,^30^^,^^53^^,^^62^^,^^64^ 3 evaluated fructans (ie, inulin, short-chain FOS, and oligofructose-enriched inulin)^29^^,^^46^^,^^63^, and 1 evaluated both GOS and FOS separately.^51^ Two trials evaluated other types of fermentable fibers, such as resistant dextrin^61^ and polydextrose (Figure S3A in the Supporting Information online).^31^ Depressive and anxiety outcomes were assessed using a variety of methods (Figure S3B in the Supporting Information online). Four RCTs assessed depressive outcomes,^30^^,^^31^^,^^61^^,^^64^ 2 RCTs assessed anxiety outcomes,^51^^,^^62^ and both depressive and anxiety outcomes were assessed in 4 RCTs.^29^^,^^46^^,^^53^^,^^63^ A range of intervention doses was evaluated, with less than half of RCTs evaluating lower dose (<5.5 g/d).^29^^,^^30^^,^^51^^,^^64^

Risk of bias varied across RCTs. Five of the 10 RCTs were deemed low risk.^30^^,^^31^^,^^61–63^ These studies included a robust randomization process and explained their intervention assignment methods and any deviations (if present), missing and outcome data assessment, and reporting results (eg, prespecified analysis plan). Because concerns with either deviation from intended intervention, outcome measurement methods, or selection of reporting results, 4 RCTs were classed as “some concerns.”^29^^,^^51^^,^^53^^,^^64^ The RCT classed as high risk^46^ did not report the randomization process and outcome measurements satisfactorily (Table S3 in the Supporting Information online).

#### The effect of fiber supplementation on depressive and anxiety outcomes

**Figure 4:**
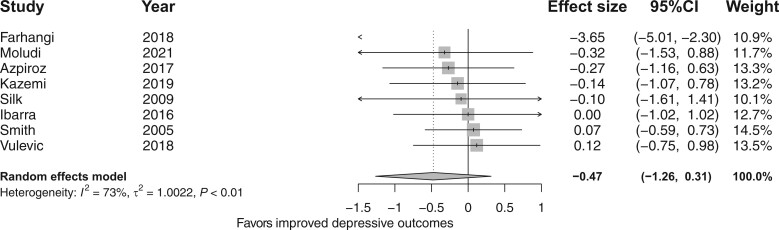
**Forest plot of randomized controlled trials investigating the effect of fiber supplementation on depressive outcomes.** Box size represents study weight and diamonds represent overall effect sizes and 95%CIs.

The meta-analysis of 8 RCTs^29–31^^,^^46^^,^^53^^,^^61^^,^^63^^,^^64^ showed that fiber supplementation did not improve depressive symptoms compared with placebo (*d* = −0.47; 95%CI, −1.26, 0.31; *I*^2^ = 73%; Figure 4).^29–31^^,^^46^^,^^53^^,^^61^^,^^63^^,^^64^ Moreover, subgroup analyses (Figure S4 in the Supporting Information online) showed that neither the risk-of-bias profiles (*x*^2^ =1.24; df = 1; *P *=* *0.27), supplementation dose (*x*^2^ = 0.81; df = 1; *P *=* *0.37) nor fiber types (*x*^2^ = 0.93; df = 2; *P *=* *0.63) (Figure S5 in the Supporting Information online) affected depressive and anxiety symptoms.

**Figure 5:**
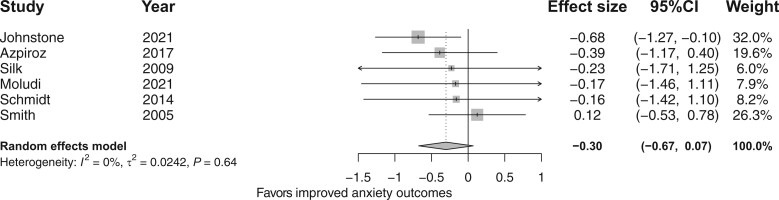
**Forest plot of randomized controlled trials investigating the effect of fiber supplementation on anxiety outcomes.** Box size represents study weight and diamonds represent overall effect sizes and 95%CIs.

The meta-analysis of 6 RCTs^29^^,^^46^^,^^51^^,^^53^^,^^62^^,^^63^ showed that fiber supplementation did not improve anxiety symptoms compared with placebo (*d* = −0.30; 95%CI, −0.67, 0.07; *I*^2^ = 0%; Figure 5).^29^^,^^46^^,^^51^^,^^53^^,^^62^^,^^63^ Moreover, subgroup analyses (Figure S6 in the Supporting Information online) showed that neither the risk-of-bias profiles (*x*^2^ = 1.88; df = 1; *P *=* *0.17) nor supplementation dose (*x*^2^ = 10.01; df = 1; *P *=* *0.92) affected depressive and anxiety outcomes. However, subgroup analysis of fiber types showed that supplementation with GOS specifically led to improved anxiety symptoms compared with placebo (*d* = −0.59; 95%CI, −1.11, −0.07; Figure S7 in the Supporting Information online).

### Publication bias and sensitivity analysis

The funnel plot generated for the meta-analyses of observational studies assessing depressive outcomes demonstrated some degree of asymmetry (Figure S8 in the Supporting Information online). This was further confirmed by the Egger’s regression test (intercept: −1.914; *P *<* *0.001). One study was identified as a potential outlier^56^ in the funnel plot. Sensitivity analysis conducted by excluding this study slightly reduced the final combined ES; however, the estimates remained significant (*d* = −0.09; 95%CI, −0.13, −0.05; Figure S9 in the Supporting Information online). Sensitivity analyses conducted by excluding cross-sectional studies with populations with disease or older people did not change the overall outcome (Figures S10–S12 in the Supporting Information online).

## DISCUSSION

To our knowledge, this systematic literature review and meta-analysis is the first to provide an exhaustive overview of the evidence for the role of dietary fiber intake on depressive and anxiety outcomes. From our analysis of observational studies, a small but significant inverse association between total fiber intake and depressive and anxiety outcomes was found. However, RCTs (which were limited in number and quality) showed that fiber supplementation was not efficacious in improving depressive or anxiety symptoms.

Our finding from cross-sectional and longitudinal studies is consistent with the findings of a previous systematic literature review and meta-analysis conducted by Fatahi et al^27^ that showed higher fiber intake was associated with reduced odds for depression; however, those authors did not assess the association between fiber intake and anxiety outcomes. In contrast, we assessed anxiety outcomes and showed an inverse association between total fiber intake and anxiety outcomes.

When considering individual studies, the relationship between total fiber intake and depressive and/or anxiety outcomes differed according to the population being studied. For example, when considering cross-sectional studies, total fiber intake was low in individuals with HIV who were depressed compared with nondepressed in individuals with HIV.^56^ In contrast there was no association between total fiber intake and depressive and anxiety outcomes in individuals with alcohol-use disorder.^22^ Moreover, findings also differed across studies that conducted analyses on population subsets (ie, sex, menopause status). One study^40^ reported an inverse association between total fiber intake and depressive outcome for male and not female participants, whereas 2 other studies found^43^^,^^44^ no association between total fiber intake and depressive and/or anxiety outcomes for either sex. These variations in findings across participant subsets are not unexpected, considering the many dietary, environmental, and host factors that affect mental health. Sensitivity analyses excluding studies of special populations (namely, individuals with alcohol-use disorder^22^ and HIV^56^; older adults^35^^,^^39^^,^^43^) did not influence the final outcome rendered by the meta-analysis (Figures S10–S12 in the Supporting Information online), indicating the overall result was not driven by studies that contained a special population. One^24^ of the 3 longitudinal studies reported no association between fiber intake and depressive or anxiety outcomes. This study differed from the others in that it measured total prebiotic fiber intake rather than total fiber intake.^24^ An FFQ was used to measure dietary intake; however, this has not been validated to measure prebiotic intake.

The meta-analytic findings from the case-control studies did not show differences in total fiber intake between depressed and nondepressed groups. However, of the 3 studies included, 2 demonstrated lower total fiber intake by depressed groups compared with the nondepressed groups.^59^^,^^60^ The study excluded from the meta-analysis^55^ also showed lower total fiber intake in the group with depression compared with the nondepressed group.

When considering RCTs, our meta-analysis showed that fiber supplementation did not improve depressive or anxiety symptoms compared with control groups. This is concordant with the findings from a smaller previous meta-analysis that particularly showed prebiotic fiber supplementation did not improve depressive or anxiety outcomes compared with the placebo.^32^ Of note, there was large variation in clinical response within studies, as evidenced by the wide CIs. Factors such as individual baseline diet including fiber intake, host health (eg, gastrointestinal conditions such as IBS), and microbiota composition may influence response to fiber supplementation.^65^ Variation in these factors may be driving the variation in clinical outcomes within studies. Identifying the individuals most likely to benefit clinically from fiber supplements is a clear area for future research.

All of the fiber interventions included in the present meta-analysis were delivered through supplementation rather than a whole-diet modification. When considering fiber intervention for improving depressive or anxiety outcomes, whole-diet modification may be more beneficial than single-fiber supplementation. This is because whole-diet modification may facilitate the increased intake of a range of fibers and thereby render broader range of effects on the gut microbiota and its metabolites,^66^ which consequently might exert a stronger influence on depressive or anxiety outcome. Measurement of adherence to interventions is critically important when interpretating results of RCTs. It is notable that of the RCTs included in this review, all except 2 measured adherence, and in 1 study, participants consumed the intervention onsite.^31^ Importantly, more than half of the studies reported an adherence rate of greater than 80%. Only 5 RCTs reported the method of monitoring adverse events,^29–31^^,^^53^^,^^61^ and none of the studies reported serious or significant adverse events related to the fiber supplementation. There were some form of mild to moderate adverse events, however, reported in a smaller proportion of individuals, suggesting that fiber supplementation was well tolerated by most of the participants. Of note, fiber supplementation trials in individuals with IBS also reported good tolerance both at higher and lower doses.^29^^,^^53^

Although fermentable fibers hold potential to influence depressive or anxiety outcomes, the type and minimum dose of fiber used to elicit improvement in depression or anxiety remain elusive. In our study, more than half of the RCTs evaluated the effect of fiber supplementation at levels defined as high in this study (>5.5 g/d) on depressive or anxiety outcomes; however, these RCTs did not provide a rationale for dose selection. Importantly, subgroup analyses showed that neither low nor high dose of prebiotic fiber supplementation had an effect on depressive or anxiety symptoms. Interestingly, subgroup analysis by fiber types showed that supplementation of GOS, but not fructans, was effective in ameliorating anxiety symptoms, compared with placebo. This might be due to the greater potential of GOS to increase fecal *Bifidobacterium* count and influence biochemical pathways underlying anxiety (eg, brain-derived neurotropic factor expression and *N*-methyl-d-aspartate receptor signalling) compared with FOS, as demonstrated in a previous animal study.^67^ However, it is worth noting that of the 3 studies included in the subgroup analysis, only 1 demonstrated a significant contribution to the overall ES; more studies are required to confirm this observation.

Our study has several strengths. We used a comprehensive search strategy and robust study design, and we undertook screening of articles, data extraction, and quality appraisal by 2 independent authors. However, there were number of limitations. First, most of the observational studies and RCTs included healthy participants rather than individuals with clinical depression or elevated depressive symptoms. This potentially limited the capacity for detecting associations or treatment effects, especially in the context that a previous meta-analysis of probiotics showed a more pronounced effect in clinical populations compared with community samples.^32^ Also, we only considered studies with an adult population aged ≥18 years; therefore, the generalizability of our findings remains limited for younger individuals. Furthermore, we were unable to perform subgroup analyses based on health status (eg, clinically diagnosed depression or anxiety vs a healthy population, or clinically diagnosed depression together with significant comorbid condition vs a healthy population) both in observational studies and RCTs, due to the smaller number of studies of clinical populations,

Second, many of the studies included did not perform a sample size or power calculation, which might have led to the risk of being underpowered to detect any statistically meaningful associations or effects, or contributed to overall smaller ESs found on meta-analyses. Specifically, only 2 cross-sectional studies^22^^,^^43^ performed sample size calculations, and although more than half of the RCTs performed a sample size calculation, only 2 RCTs based the power calculation on depressive and/or anxiety outcomes.^30^^,^^63^

Third, there were limitations in the methods used to assess the exposure and outcome. With regard to fiber intake, several self-reported questionnaires were used in observational studies, all of which are known to be prone to some error, particularly recall bias and the Hawthorne effect. However, some methods, such as FFQs, which were used by a majority of the studies, are more prone to overreporting error.^68^ Furthermore, some studies used dietary assessment methods that had little or no validation. In a similar vein, most of the observational studies and RCTs used several self-reported questionnaires, which are prone to reporting bias, to assess for depressive and anxiety outcomes. Moreover, the use of a variety of methods may have contributed to the variability in findings.

Finally, there were several limitations with relation to the study designs of observational studies and RCTs. Most of the observational studies had a cross-sectional study design, which inherently lacks evidence for temporality. Although the temporality issue is avoided in longitudinal and case-control study designs, they are prone to recall bias.^69^ Moreover, there was higher heterogeneity in the cross-sectional meta-analytic findings between total fiber intake and depressive outcomes (*I*^2^ = 74%). This might relate to the variation among studies included, including a wide range of geographic locations of participants and variable participant age range. Furthermore, there were inconsistencies in confounders or covariates used for statistical model adjustments across observational studies. Although most of the studies accounted for key factors such as age, body mass index, and smoking (Table 2), none of the studies accounted for diet quality, which may have led to residual confounding. RCTs are considered the gold standard for assessing causal effects; however, of the included trials, depressive or anxiety outcomes were not included as primary end points in a majority of RCTs. In addition, short trial duration (2–8 weeks) may have contributed to a lack of significant findings. Comparatively, RCTs of antidepressant therapy span across 6–12 weeks.^70^^,^^71^

## CONCLUSION

In summary, our study showed a beneficial but modest association between total fiber intake and depressive and anxiety outcomes in observational studies; however, because of limitations in sampling factors such as population health status and age, the generalizability of our finding remains limited. On the other hand, findings from RCTs did not show an overall beneficial effect of fiber supplementation on improving depressive or anxiety symptoms compared with placebo in a generally healthy population, although there may be potential for GOS in improving anxiety symptoms. Adequately powered RCTs, with robust trial design, of a broader range of well-defined fibers including whole-diet interventions in clinical populations are warranted.

## Supplementary Material

nuad143_Supplementary_Data

## Supporting Information

The following Supporting Information is available through the online version of this article at the publisher’s website.

**Search string**

*Table S1* **Statistical estimates used in observational studies and randomized controlled trials**

*Table S2* **The association between different types of fiber and depressive or anxiety outcomes in observational studies**

*Table S3* **Risk-of-bias assessment for observational studies**

*Table S4* **Risk-of-bias assessment for randomized controlled trials**

*Figure S1* **Exposure and outcome assessment methods used in observational studies by study design**

*Figure S2* **Forest plot of cross-sectional studies investigating the association between fiber intake and depressive outcomes sub-grouped by risk of bias categories**

*Figure S3* **Fiber supplementation types and outcome assessment methods used in randomized controlled trials**

*Figure S4* **Forest plot of randomized controlled trials investigating the effect of fiber supplementation on depressive outcomes sub-grouped by risk of bias categories and dose**

*Figure S5* **Forest plot of randomized controlled trials investigating the effect of fiber supplementation on depressive outcomes sub-grouped by fiber types**

*Figure S6* **Forest plot of randomized controlled trials investigating the effect of fiber supplementation on anxiety outcomes sub-grouped by risk of bias categories and dose**

*Figure S7* **Forest plot of randomized controlled trials investigating the effect of fiber supplementation on anxiety outcomes sub-grouped by fiber types**

*Figure S8* **Funnel plot for depressive outcomes in observational studies**

*Figure S9* **Forest plot for the meta-analysis conducted by excluding Purnomo et al**^56^**(sensitivity analysis) for depressive outcome in observational studies**

*Figure S10* **Forest plot for the meta-analysis conducted by excluding studies with clinical population (ie, Amadieu et al**^22^**and Purnomo et al**^56^**) for depressive outcome in observational studies (sensitivity analysis)**

*Figure S11* **Forest plot for the meta-analysis conducted by excluding studies with an older population (ie, Chrzastek et al,**^43^**Gopinath et al,**^35^**and Woo et al**^39^**) for depressive outcome in observational studies (sensitivity analysis)**

*Figure S12* **Forest plot for the meta-analysis conducted by excluding studies with both clinical and older population (ie, Amadieu et al,**^22^**Purnomo et al,**^56^**Chrzastek et al,**^43^**Gopinath et al,**^35^**and Woo et al**^39^**) for depressive outcome in observational studies (sensitivity analysis)**
