# Supplementary material for: Fiber intake and fiber intervention in depression and anxiety: a systematic review and meta-analysis of observational studies and randomized controlled trials
Source: Nutr Rev. 2023 Nov 25;82(12):1678–95. doi: 10.1093/nutrit/nuad143 (PMC11551482; doi:10.1093/nutrit/nuad143)
Supplement: nuad143_Supplementary_Data [file nuad143_supplementary_data.zip › nuad143_Supplementary_Data/Appendix_S1.pdf]

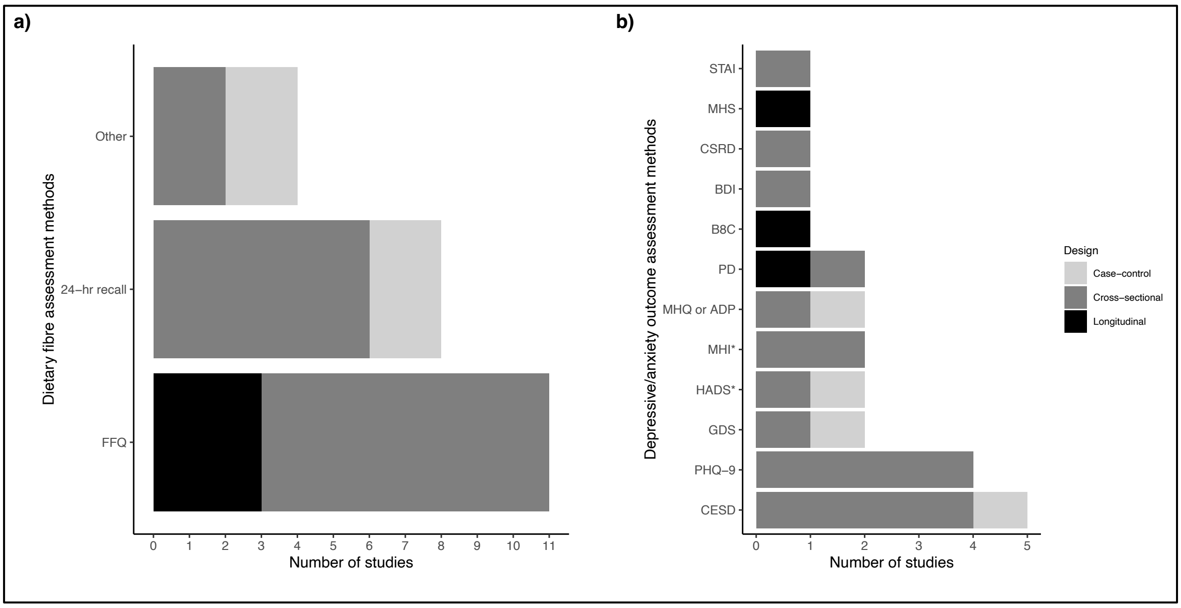

Supplemental Figure 1. Exposure and outcome assessment methods used in observational studies by study design**.** a) Dietary fibre intake assessment methods b) Depressive and anxiety outcome assessment methods. FFQ: food frequency questionnaire and other includes feeding diet history questionnaires and 3-day recall; STAI: state-trait anxiety inventory (anxiety assessment); MHS: mental health score; CSRD: Chinese version of the Zung Self-Rating Depression Scale; BDI: Beck depression inventory; B8C: Burnam 8-item scale; PD: Physician diagnosed; MHQ or ADP: use of a mental health questionnaire or anti-depressant; MHI: Mental health interview; HADS: Hospital anxiety and depression scale; GDS: geriatric depression scale; PHQ-9: patient health questionnaire; CESD: centre for epidemiologic studies depression; *on MHI and HADS denotes that these methods were used to assess anxiety measures as well.

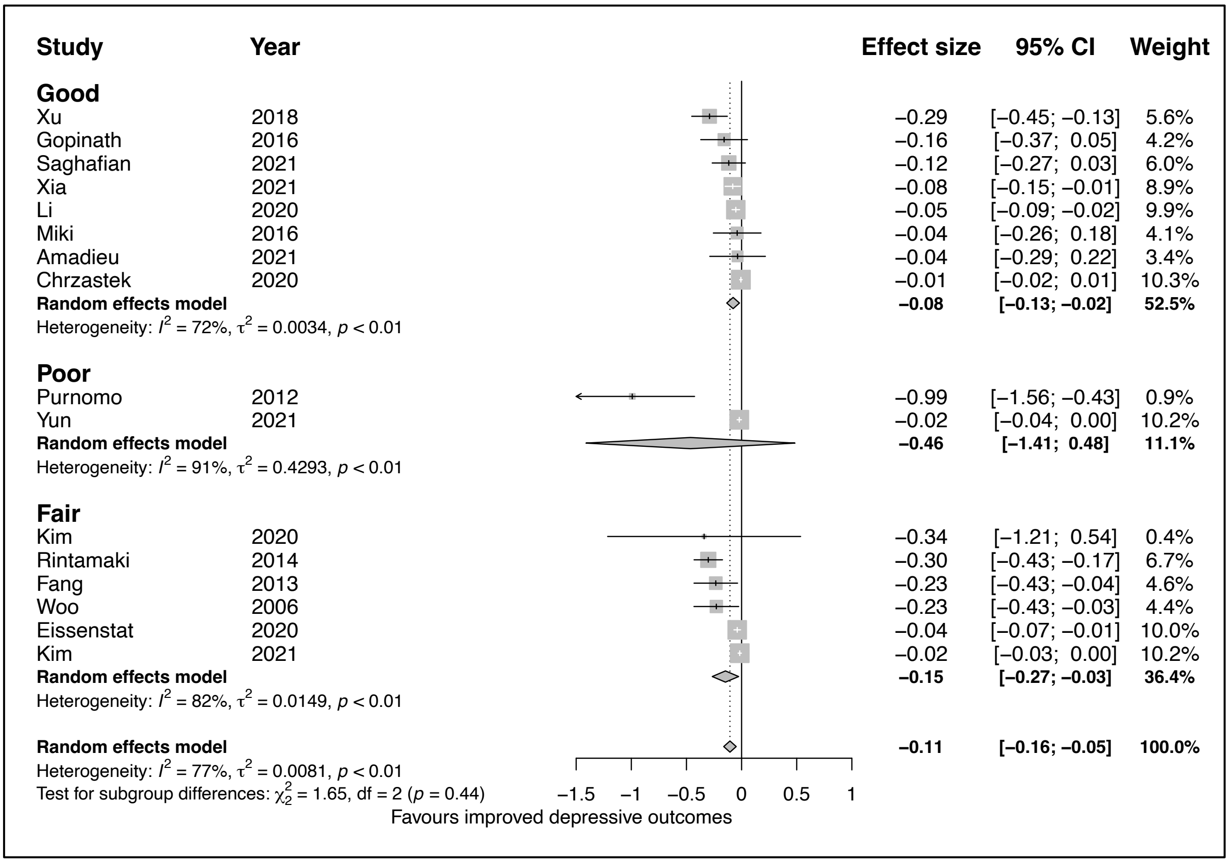

Supplemental Figure 2. Forest plot of cross-sectional studies investigating the association between fibre intake and depressive outcomes sub-grouped by risk of bias categories

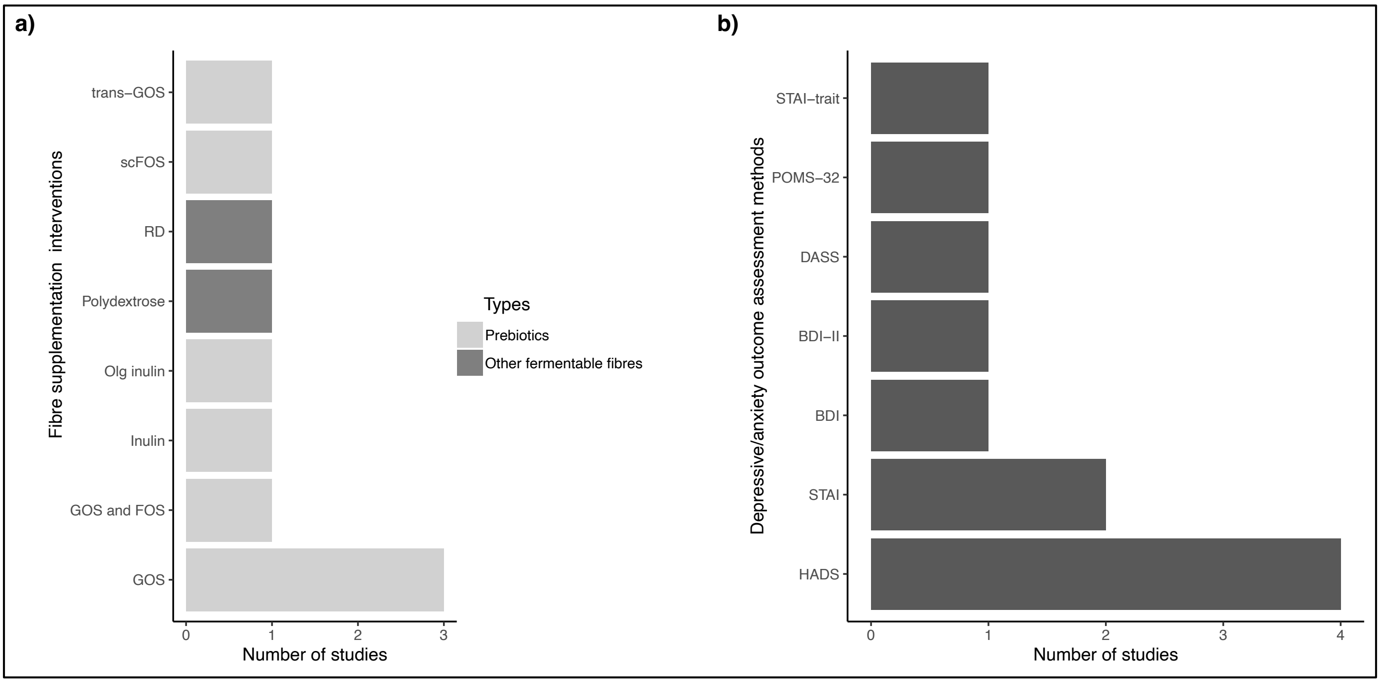

Supplemental Figure 3. Fibre supplementation types and outcome assessment methods used in randomised controlled trials. a) Types of fibre supplementations evaluated b) Depressive and anxiety outcome assessment methods. GOS: galacto-oligosaccharide; scFOS: short chain fructo-oligosaccharide; RD: resistant dextrin; Olg inulin: oligo fructose enriched inulin; STAI: state-trait anxiety inventory (anxiety assessment); POMS: profile of mood states questionnaire; DASS: depression anxiety stress scale; BDI: Beck depression inventory; HADS: Hospital anxiety and depression scale

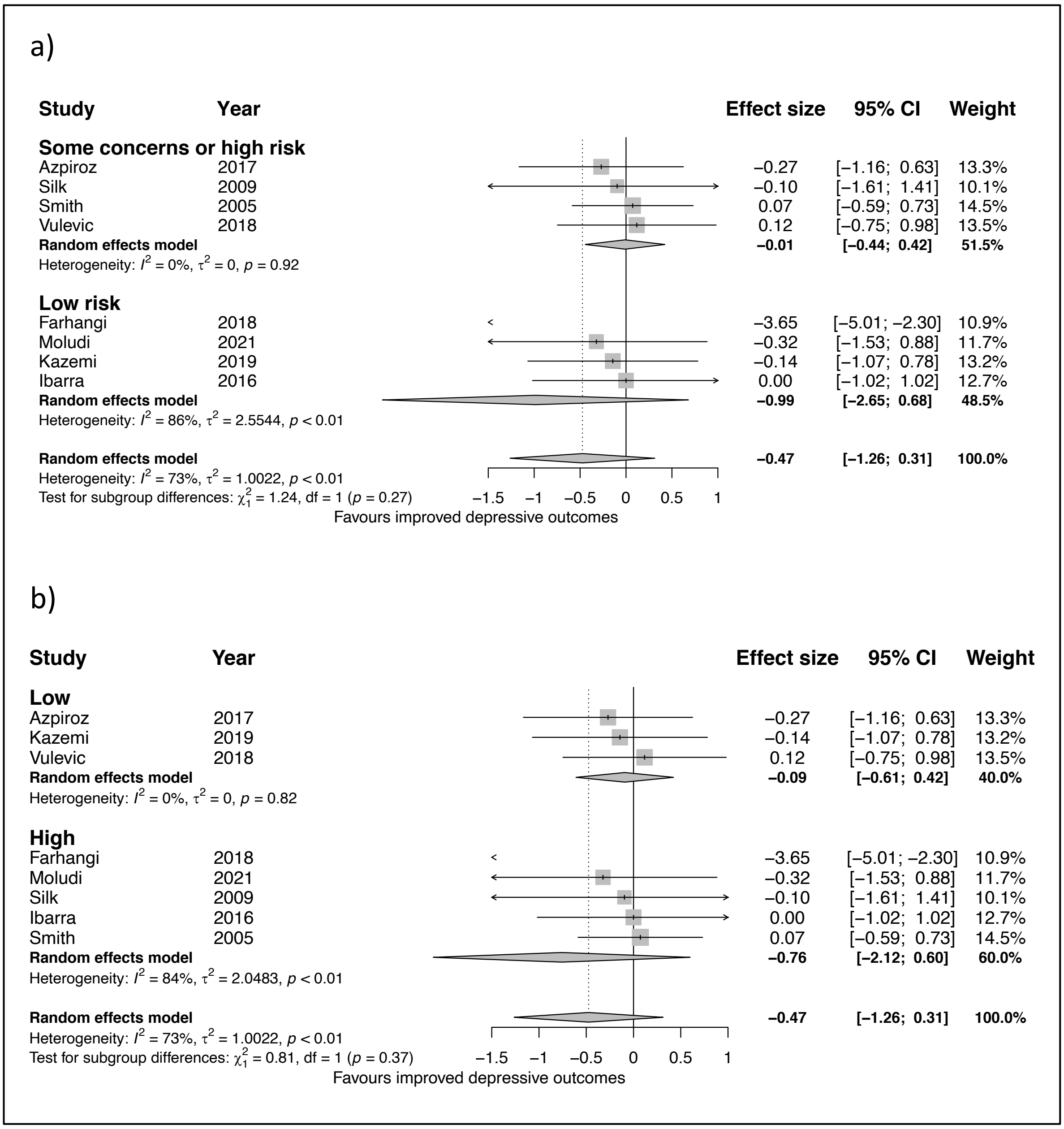

Supplemental Figure 4. Forest plot of randomised controlled trials investigating the effect of fibre supplementation on depressive outcomes sub-grouped by a) risk of bias categories: low-risk vs some concerns or high-risk and b) dose: low (≤5.5 g/d) vs high: (>5.5 g/d).

- When sub-grouping risk of bias categories (low, some concerns or high risk), the risk categories "some concerns" and "high" were combined together as only one study reported high risk

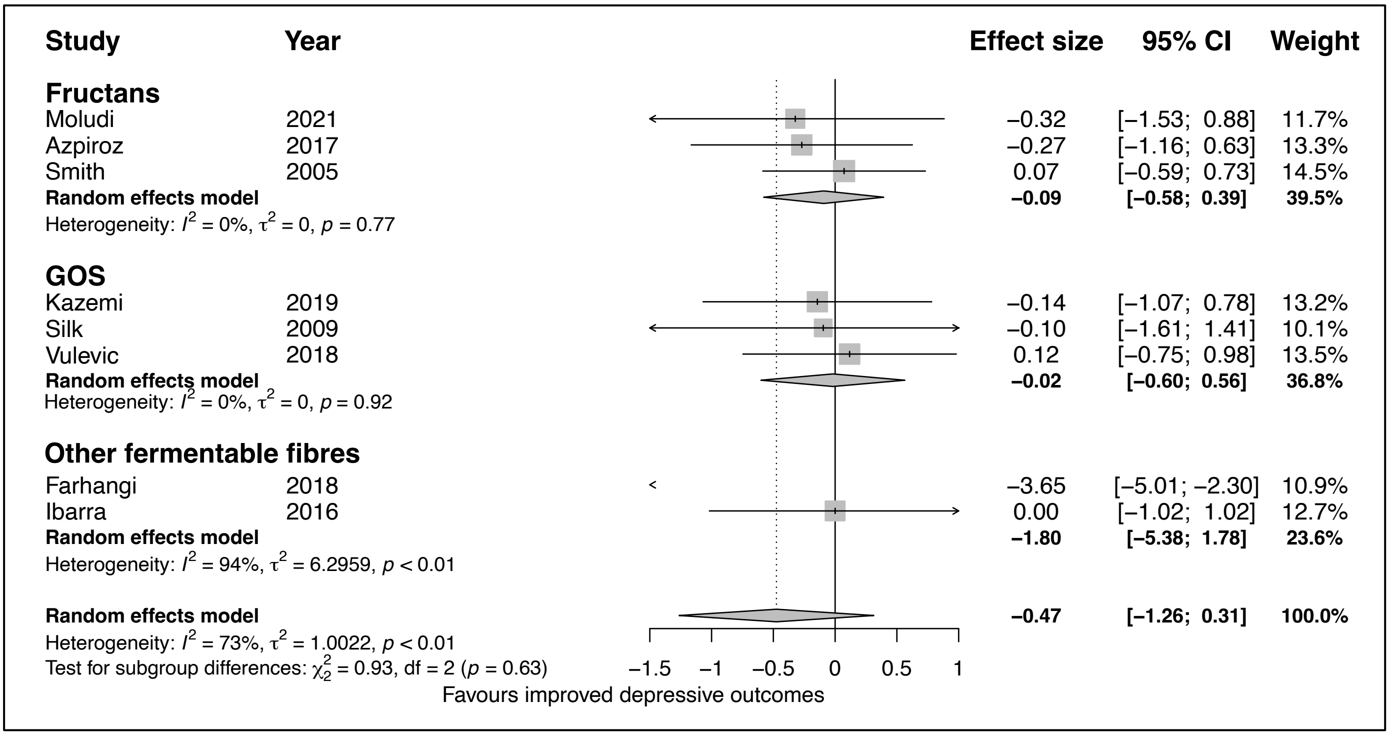

Supplemental Figure 5. Forest plot of randomised controlled trials investigating the effect of fibre supplementation on depressive outcomes sub-grouped by fibre types. GOS: galacto-oligosacharide.

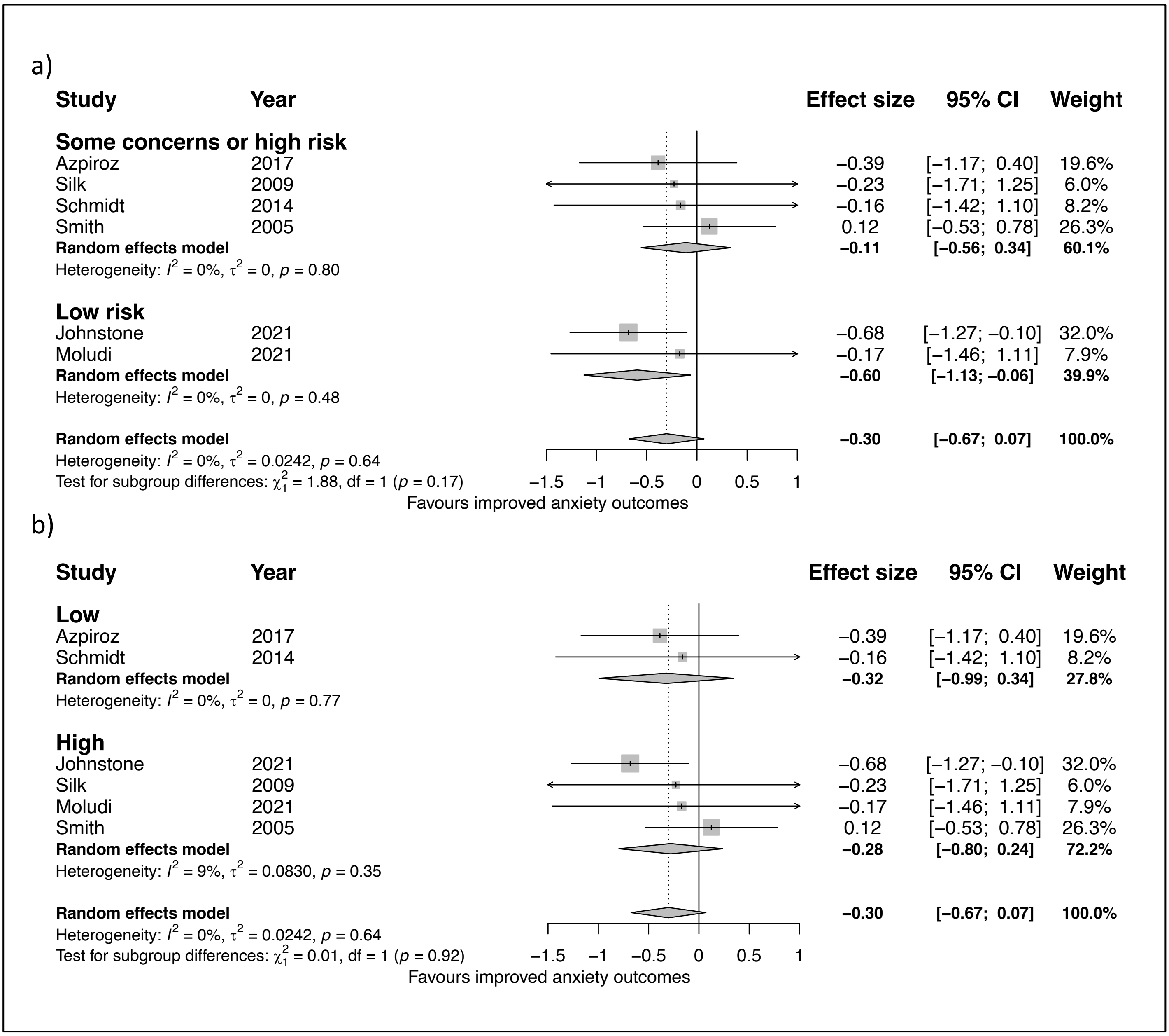

Supplemental Figure 6. Forest plot of randomised controlled trials investigating the effect of fibre supplementation on anxiety outcomes sub-grouped by a) risk of bias categories: low risk vs some concerns or high-risk and b) dose: low (≤5.5 g/d) vs high (>5.5 g/d).

- When sub-grouping risk of bias categories (low, some concerns or high risk), the risk categories "some concerns" and "high" were combined together as only one study reported high risk

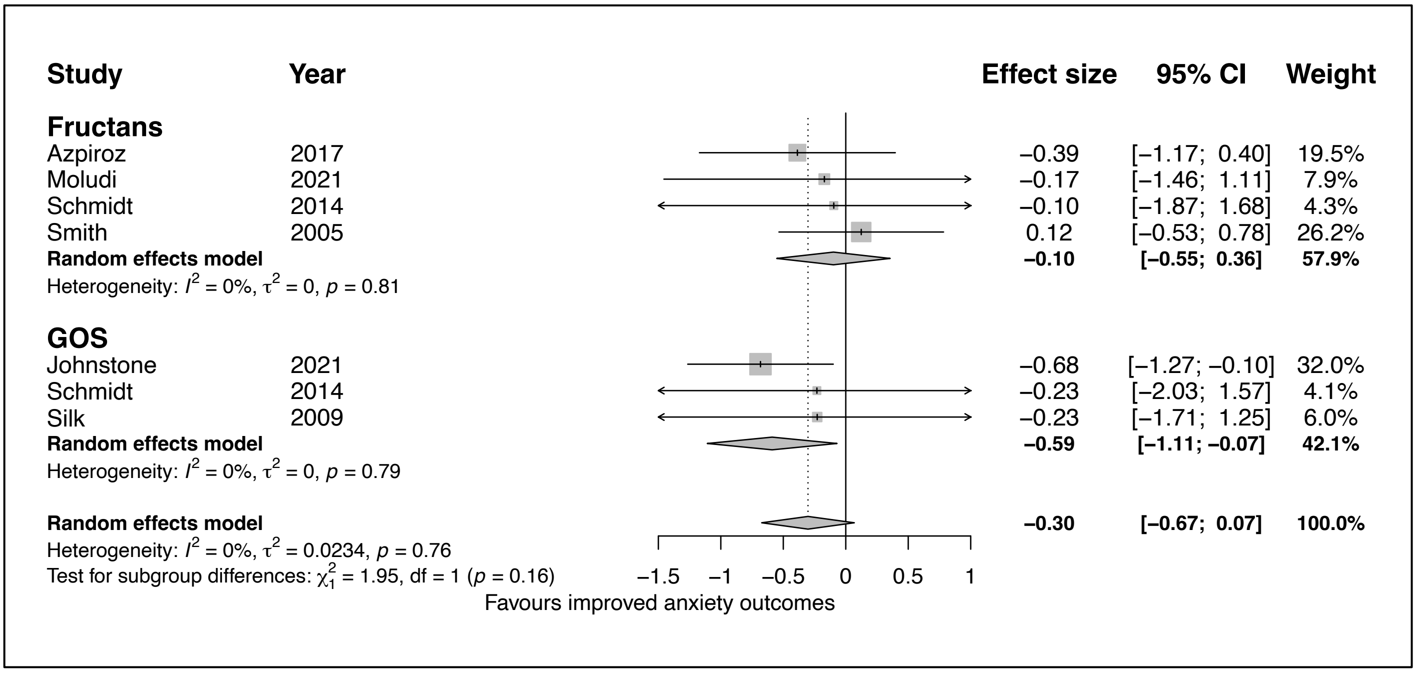

Supplemental Figure 7. Forest plot of randomised controlled trials investigating the effect of fibre supplementation on anxiety outcomes sub-grouped by fibre types. GOS: galacto-oligosacharide

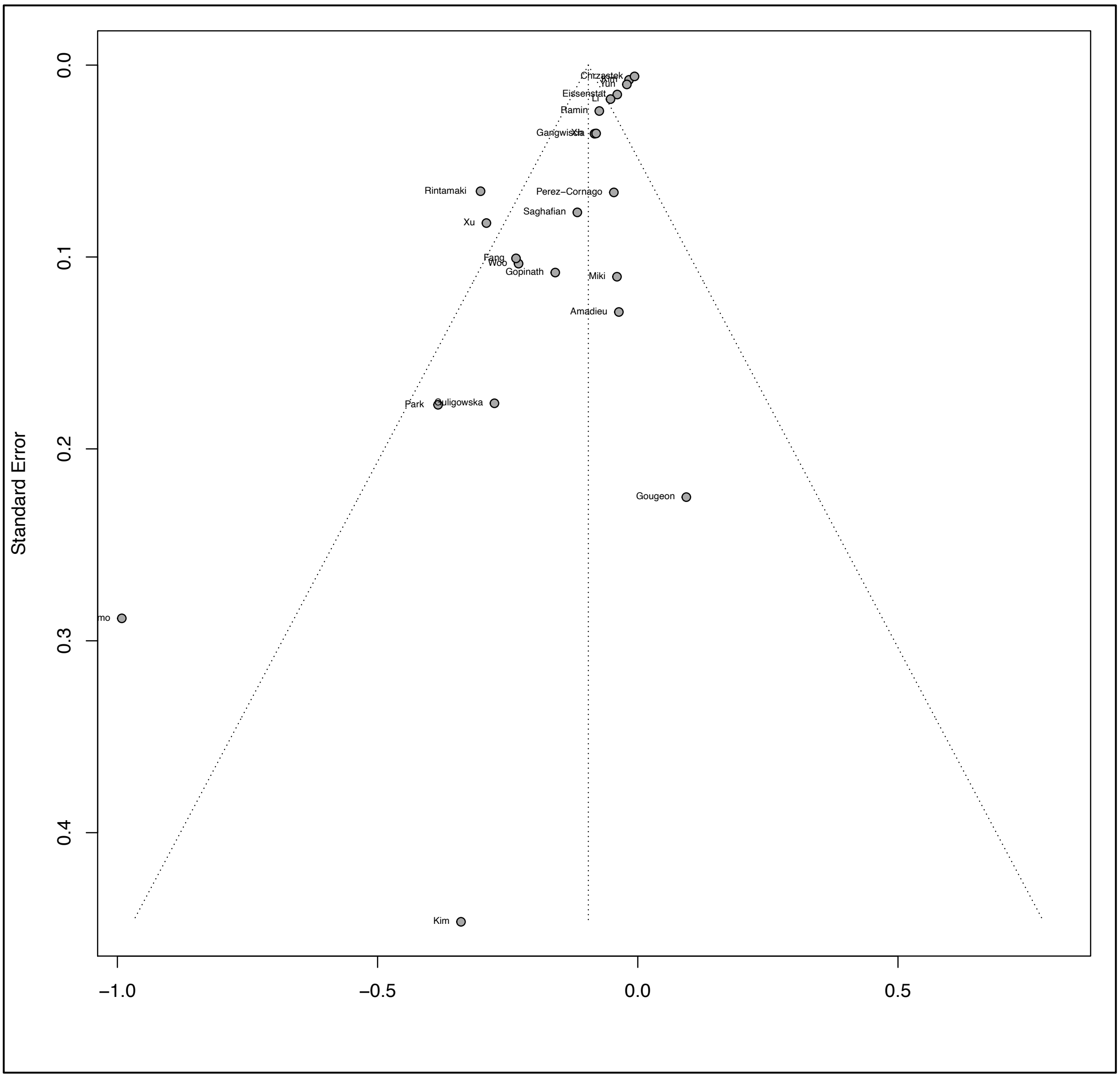

Supplemental Figure 8. Funnel plot for depressive outcomes in observational studies

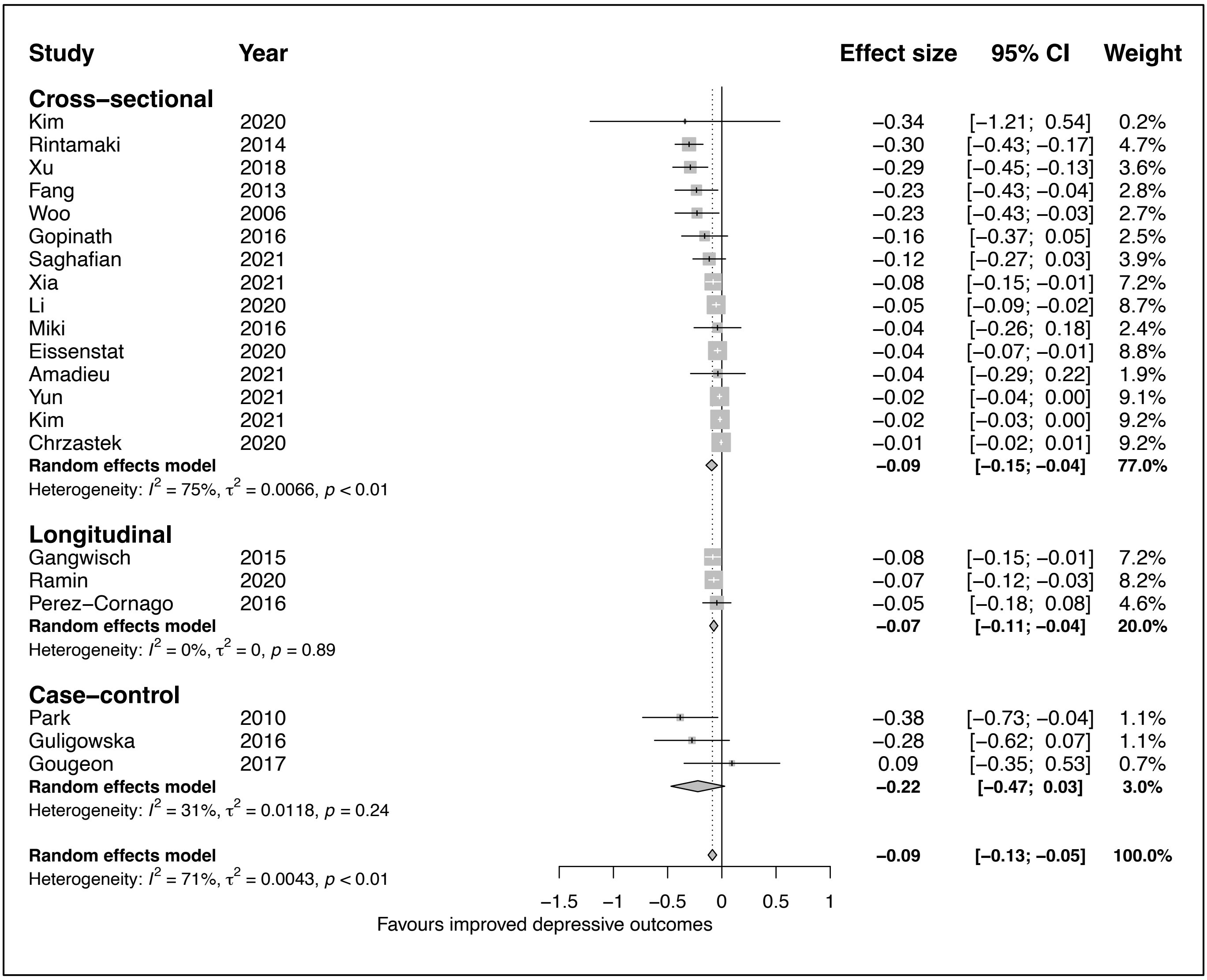

Supplemental Figure 9. Forest plot for the meta-analysis conducted by excluding Purnomo et al., (sensitivity analysis) for depressive outcome in observational studies. Box size represents study weight and diamonds represent overall effect sizes and 95% CI for each pooled study type and overall pooled results.

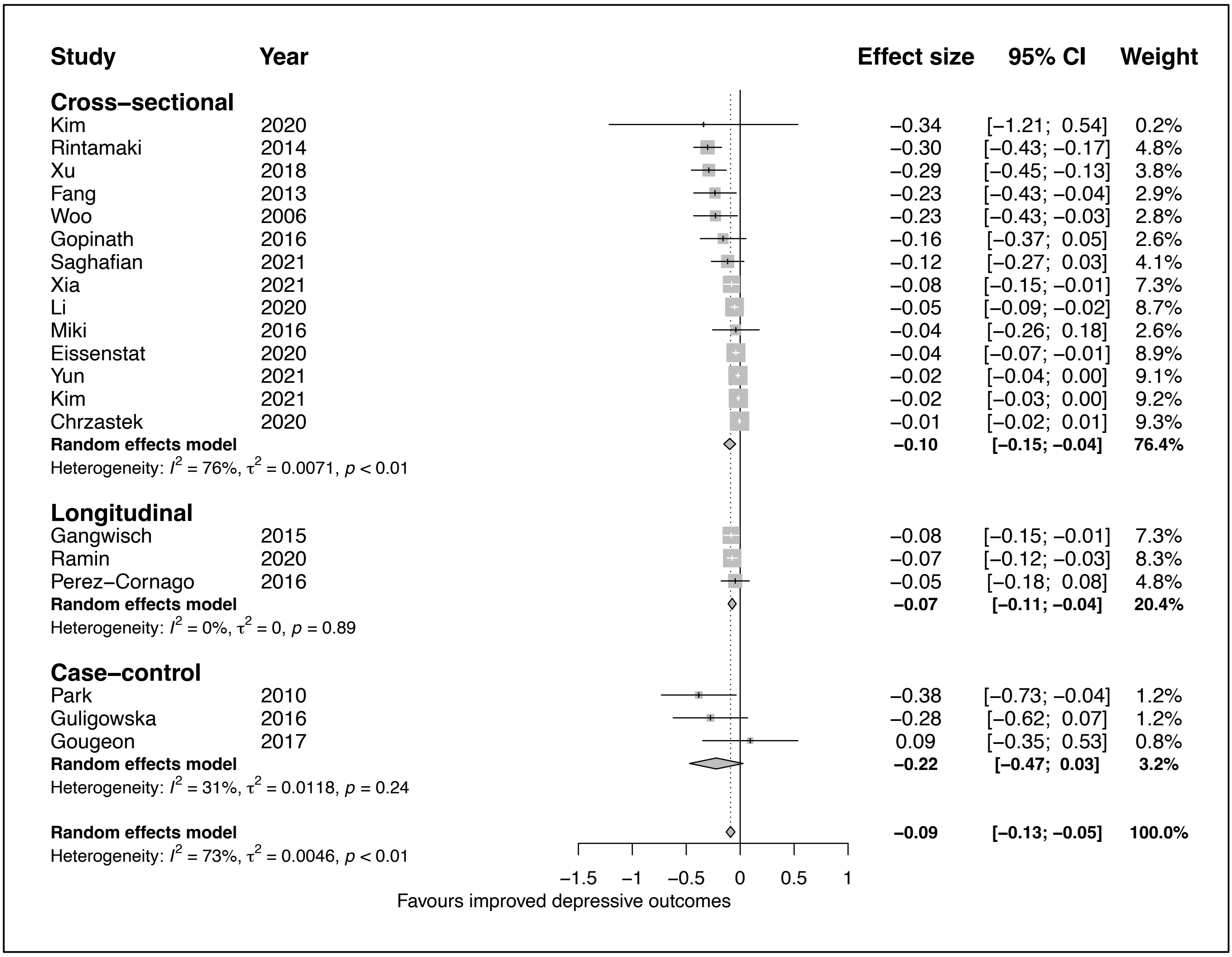

Supplemental Figure 10. Forest plot for the meta-analysis conducted by excluding studies with clinical population (i.e., Amadieu et al, Purnomo et al) for depressive outcome in observational studies (sensitivity analysis). Box size represents study weight and diamonds represent overall effect sizes and 95% CI for each pooled study type and overall pooled results.

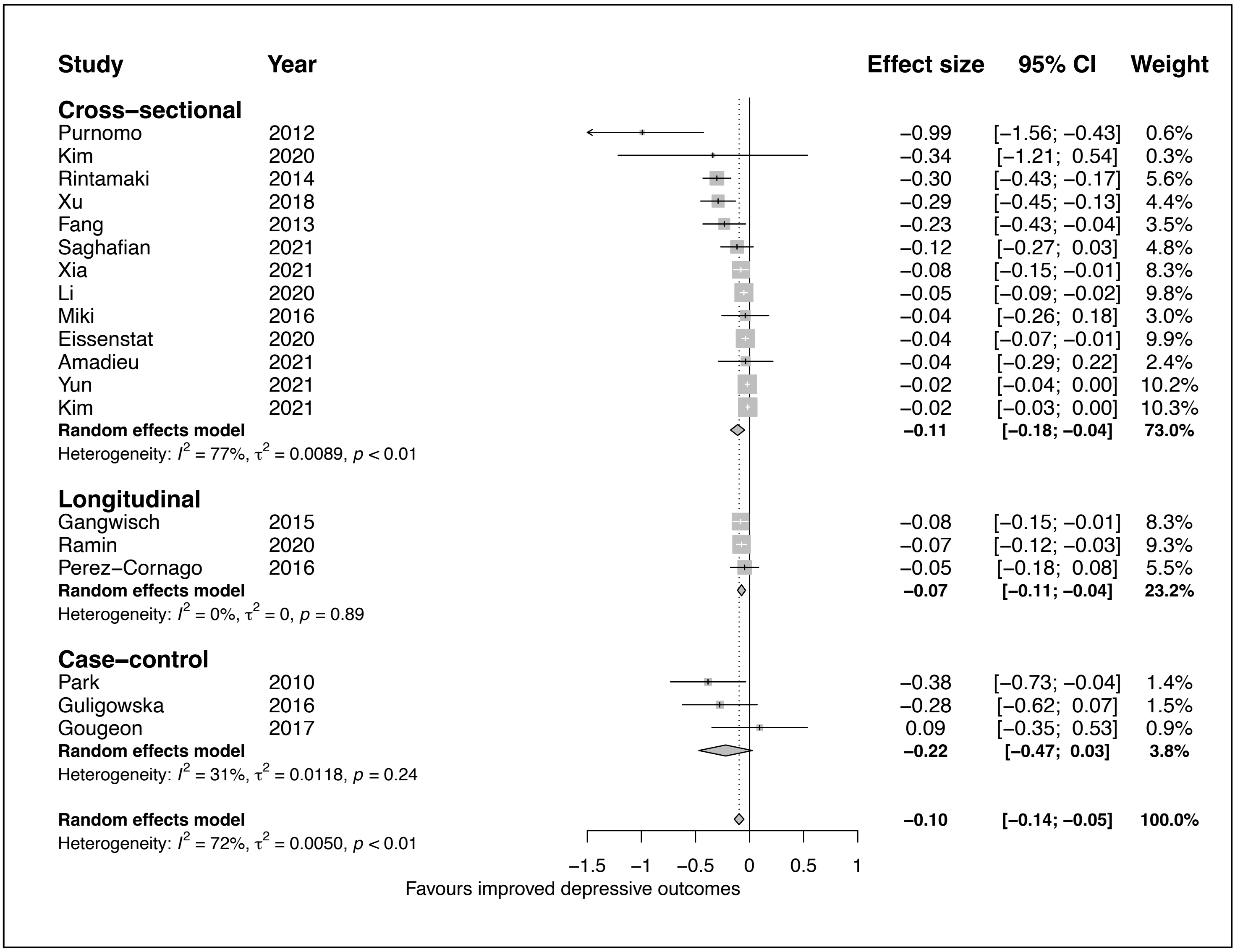

Supplemental Figure 11. Forest plot for the meta-analysis conducted by excluding studies with an older population (i.e., Chrzastek et al, Gopinath et al, Woo et al) for depressive outcome in observational studies (sensitivity analysis). Box size represents study weight and diamonds represent overall effect sizes and 95% CI for each pooled study type and overall pooled results.

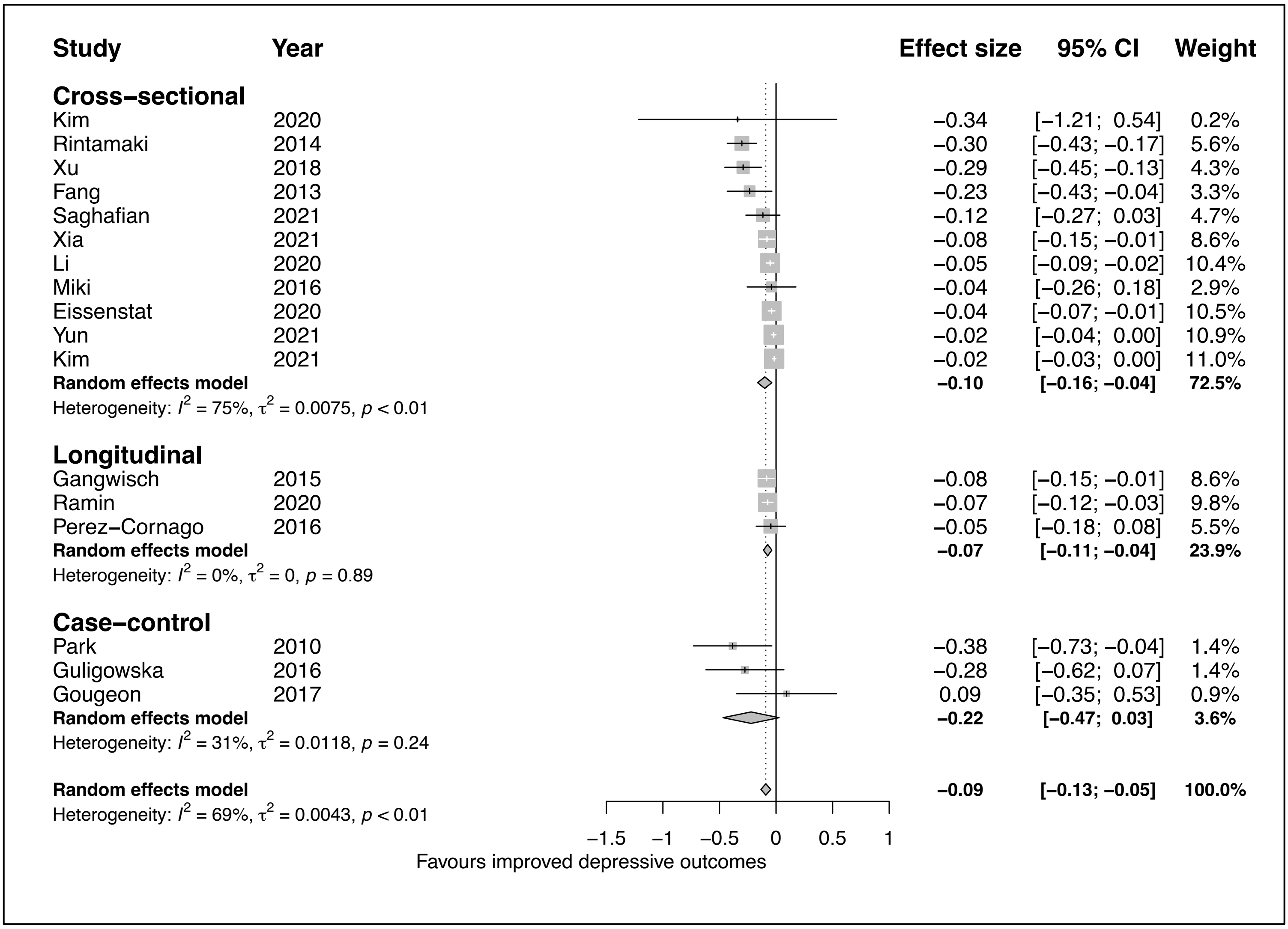

Supplemental Figure 12. Forest plot for the meta-analysis conducted by excluding studies with both clinical and older population (i.e., Amadieu et al, Purnomo et al, Chrzastek et al, Gopinath et al, Woo et al) for depressive outcome in observational studies (sensitivity analysis). Box size represents study weight and diamonds represent overall effect sizes and 95% CI for each pooled study type and overall pooled results.

Supplemental Table 1. Statistical estimates used in observational studies and randomised controlled trials

Observational studies

	Study	Study design	Exposure variable (continuous/categorical)	Outcome variable (continuous/categorical)	Statistical estimates
Cross-sectional	Amadieu et al[S1]	Linear regression	Continuous	Continuous	β and 95% CI
	Chrzastek et al[S2]	Logistic regression	Continuous	Categorical/dichotomised	OR and 95% CI
	Eissenstat et al[S3]	Linear regression	Continuous	Continuous	β and t
	Fang et al [S4]	Linear regression	Continuous	Continuous	β and t
	Gopinath et al [S5]	Logistic regression	Categorical (tertile)	Categorical/dichotomised	OR, 95% CI
	Kim et al [S6]	Logistic regression	Categorical (quartiles)	Categorical/dichotomised	OR, 95% CI
	Kim et al [S7]	Logistic regression	Continuous	Categorical/dichotomised	OR, 95% CI
	Li et al [S8]	Linear regression	Continuous	Continuous	β , 95% CI
	Miki et al [S9]	Logistic regression	Categorical (tertile)	Categorical/dichotomised	OR, 95% CI
	Purnomo et al [S10]	Mann-Whitney U test	Continuous	Categorical/dichotomised	Mean and SD
	Rintamaki et al [S11]	General linear model	Continuous	Categorical/dichotomised	Mean and 95% CI
	Saghafian et al [S12]	Logistic regression	Categorical (quartiles)	Categorical/dichotomised	OR, 95% CI
	Woo et al [S13]	Logistic regression	Categorical (tertile)	Categorical/dichotomised	OR, 95% CI
	Xia et al [S14]	Logistic regression	Categorical (quartiles)	Categorical/dichotomised	OR, 95% CI
	Xu et al [S15]	Logistic regression	Categorical (quartiles)	Categorical/dichotomised	OR, 95% CI
	Yun et al [S16]	Linear regression	Continuous	Continuous	β and 95% CI
Longitudinal	Gangwisch [S17]	Logistic regression	Categorical (quintiles)	Categorical/dichotomised	OR and 95% CI
	Perez-Cornago [S18]	Multivariable Cox proportional hazard analysis	Categorical (quartiles)	Categorical/dichotomised	HR and 95% CI
	Ramin et al [S19]	Linear regression	Categorical (quartiles)	Continuous	β and 95% CI
Case-control	Gougeon et al [S20]	General linear model	Continuous	Categorical/dichotomised	Mean and SD
	Guligowska et al [S21]	Mann-Whitney U test	Continuous	Categorical/dichotomised	Mean and SD
	*Othman et al [S22]	Student's T- test	Continuous	Categorical/dichotomised	Mean
	Park et al [S23]	Student's T- test	Continuous	Categorical/dichotomised	Mean and SE

Randomised Controlled Trials

	Study design	Reported statistical estimates
Azpiroz et al [S24]	Randomized, double-blind, parallel, placebo-controlled trial	Mean and SD
Farhangi et al [S25]	Randomized, triple-blind, placebo-controlled clinical trial	Mean and SD
Ibarra et al [S26]	Acute, randomized, double-blind, four-arm crossover, placebo-controlled trial	Mean and SD
Johnstone et al [S27]	Randomized, double-blind, placebo-controlled trial	*f* and *p* value
Kazemi et al [S28]	Randomized, double-blind, three-arm parallel, placebo-controlled trial	Mean and 95% CI
Moludi et al [S29]	Randomized, double-blind, four-arm parallel, placebo-controlled trial	Mean and SD
Schmidt et al [S30]	Randomised, double-blind, placebo-controlled trial	Mean and SD
Silk et al [S31]	Randomized, blinded, cross over, parallel, placebo-controlled trial	Mean and SD
Vulevic et al [S32]	Randomized, double-blind, crossover, placebo-controlled trial	Mean and SD
Smith et al [S33]	Randomised, controlled, cross over placebo-controlled trial	Mean and SE

β: beta-coefficient; *t*: t statistics; OR: odds ratio; CI: confidence interval; SD: standard deviation; SE: standard error; HR: hazard ratio; *f* statistics:

* this study was not included in meta-analysis due to not having the required level of information for analysis.

References:

S1. Amadieu C, Leclercq S, Coste V, et al. Dietary fiber deficiency as a component of malnutrition associated with psychological alterations in alcohol use disorder. *Clin Nutr*. 2021;40(5):2673-2682.

S2. Chrzastek Z, Guligowska A, Piglowska M, Soltysik B, Kostka T. Association between sucrose and fiber intake and symptoms of depression in older people. *Nutr Neurosci*. 2022;25(5):886-897.

S3. Eissenstat SJ, Gao N, Radler D, Oh TL. Nutrient intake differences among ethnic groups and risks of depression. *Journal of Immigrant and Minority Health*. 2020;22(6):1141-1148.

S4. Fang CY, Egleston BL, Gabriel KP, et al. Depressive symptoms and serum lipid levels in young adult women. *J Behav Med*. 2013;36(2):143-152

S5. Gopinath B, Flood VM, Burlutksy G, Louie JC, Mitchell P. Association between carbohydrate nutrition and prevalence of depressive symptoms in older adults. *Br J Nutr*. 2016;116(12):2109-2114

S6. Kim C-S, Byeon S, Shin D-M. Sources of dietary fiber are differently associated with prevalence of depression. *Nutrients*. 2020;12(9):2813

S7. Kim Y, Hong M, Kim S, Shin W-y, Kim J-h. Inverse association between dietary fiber intake and depression in premenopausal women: a nationwide population-based survey. *Menopause*. 2021;28(2):150-156.

S8. Li D, Tong Y, Li Y. Dietary fiber is inversely associated with depressive symptoms in premenopausal women. *Front Neurosci*. 2020;14:373.

S9. Miki T, Eguchi M, Kurotani K, et al. Dietary fiber intake and depressive symptoms in Japanese employees: The Furukawa Nutrition and Health Study. *Nutrition*. 2016;32(5):584-589.

S10. Purnomo J, Jeganathan S, Begley K, Houtzager L. Depression and dietary intake in a cohort of HIV-positive clients in Sydney. *Int J STD AIDS*. 2012;23(12):882-886.

S11. Rintamäki R, Kaplas N, Männistö S, et al. Difference in diet between a general population national representative sample and individuals with alcohol use disorders, but not individuals with depressive or anxiety disorders. *Nordic journal of psychiatry*. 2014;68(6):391-400

S12. Saghafian F, Sharif N, Saneei P, et al. Consumption of dietary fiber in relation to psychological disorders in adults. *Frontiers in Psychiatry*. 2021:926.

S13. Woo J, Lynn H, Lau W, et al. Nutrient intake and psychological health in an elderly Chinese population. *International Journal of Geriatric Psychiatry: A journal of the psychiatry of late life and allied sciences*. 2006;21(11):1036-1043.

S14. Xia Y, Liu Y, Zhang S, et al. Associations between different types and sources of dietary fibre intake and depressive symptoms in a general population of adults: a cross-sectional study. *Br J Nutr*. 2021;125(11):1281-1290.
S15. Xu H, Li S, Song X, Li Z, Zhang D. Exploration of the association between dietary fiber intake and depressive symptoms in adults. *Nutrition*. 2018;54:48-53.
S16. Yun H, Kim D-W, Lee E-J, Jung J, Yoo S. Analysis of the effects of nutrient intake and dietary habits on depression in Korean adults. *Nutrients*. 2021;13(4):1360.
S17. Gangwisch JE, Hale L, Garcia L, et al. High glycemic index diet as a risk factor for depression: analyses from the Women's Health Initiative. *The American journal of clinical nutrition*. 2015;102(2):454-463.
S18. Perez-Cornago A, Sanchez-Villegas A, Bes-Rastrollo M, et al. Intake of high-fat yogurt, but not of low-fat yogurt or prebiotics, is related to lower risk of depression in women of the SUN cohort study. *The Journal of nutrition*. 2016;146(9):1731-1739.
S19. Ramin S, Mysz MA, Meyer K, Capistrant B, Lazovich D, Prizment A. A prospective analysis of dietary fiber intake and mental health quality of life in the Iowa Women's Health Study. *Maturitas*. 2020;131:1-7.
S20. Gougeon L, Payette H, Morais JA, Gaudreau P, Shatenstein B, Gray-Donald K. A prospective evaluation of the depression–nutrient intake reverse causality hypothesis in a cohort of community-dwelling older Canadians. *Br J Nutr*. 2017;117(7):1032-1041.
S21. Guligowska A, Pigłowska M, Fife E, et al. Inappropriate nutrients intake is associated with lower functional status and inferior quality of life in older adults with depression. *Clin Interv Aging*. 2016;11:1505.
S22. Ben Othman R, Mziou O, Gamoudi A, et al. Nutritional Status of Depressive Patients. *J Diabetes Metab*. 2018;9(2)
S23. Park J-Y, You J-S, Chang K-J. Dietary taurine intake, nutrients intake, dietary habits and life stress by depression in Korean female college students: a case-control study.*J Biomed Sci*. 2010;17(1):1-5.
S24. Azpiroz F, Dubray C, Bernalier-Donadille A, et al. Effects of sc FOS on the composition of fecal microbiota and anxiety in patients with irritable bowel syndrome: A randomized, double blind, placebo controlled study. *Neurogastroenterol Motil*. 2017;29(2):e12911.
S25. Farhangi MA, Javid AZ, Sarmadi B, Karimi P, Dehghan P. A randomized controlled trial on the efficacy of resistant dextrin, as functional food, in women with type 2 diabetes: Targeting the hypothalamic–pituitary–adrenal axis and immune system. *Clin Nutr*. 2018;37(4):1216-1223.
S26. Ibarra A, Olli K, Pasman W, et al. Effects of polydextrose with breakfast or with a midmorning preload on food intake and other appetite-related parameters in healthy normal-weight and overweight females: An acute, randomized, double-blind, placebo-controlled, and crossover study. *Appetite*. 2017;110:15-24.
S27. Johnstone N, Milesi C, Burn O, et al. Anxiolytic effects of a galacto-oligosaccharides prebiotic in healthy females (18–25 years) with corresponding changes in gut bacterial composition. *Sci Rep*. 2021;11(1):1-11.
S28. Kazemi A, Noorbala AA, Azam K, Eskandari MH, Djafarian K. Effect of probiotic and prebiotic vs placebo on psychological outcomes in patients with major depressive disorder: A randomized clinical trial. *Clin Nutr*. 2019;38(2):522-528.
S29. Moludi J, Khedmatgozar H, Nachvak SM, Abdollahzad H, Moradinazar M, Sadeghpour Tabaei A. The effects of co-administration of probiotics and prebiotics on chronic inflammation, and depression symptoms in patients with coronary artery diseases: a randomized clinical trial. *Nutr Neurosci*. 2022;25(8):1659-1668.
S30. Schmidt K, Cowen PJ, Harmer CJ, Tzortzis G, Errington S, Burnet PW. Prebiotic intake reduces the waking cortisol response and alters emotional bias in healthy volunteers. *Psychopharmacology*. 2015;232(10):1793-1801.
S31. Silk D, Davis A, Vulevic J, Tzortzis G, Gibson G. Clinical trial: the effects of a trans-galactooligosaccharide prebiotic on faecal microbiota and symptoms in irritable bowel syndrome. *Aliment Pharmacol Ther*. 2009;29(5):508-518.
S32. Vulevic J, Tzortzis G, Juric A, Gibson GR. Effect of a prebiotic galactooligosaccharide mixture (B-GOS®) on gastrointestinal symptoms in adults selected from a general population who suffer with bloating, abdominal pain, or flatulence. *Neurogastroenterol Motil*. 2018;30(11):e13440.
S33. Smith AP. The concept of well-being: relevance to nutrition research. *Br J Nutr*. 2005;93(S1):S1-S5.

Supplemental Table 2. The association between different types of fibre and depressive or anxiety outcomes in observational studies

Author (year)	Other types of fibre assessed	Results
Amadieu et al (2021) [S1]	SDF	No association between SDF intake and depression or anxiety.
	IDF	No association between SDF intake and depression or anxiety.
	Fructans (FOS+GOS)	Inverse association between fructan intake and anxiety but not depression.
Fang et al (2013) [S2]	IDF	Inverse association between IDF intake and depression.
	SDF	No association between SDF intake and depression.
Gopinath et al (2016) [S3]	Vegetable fibre	Inverse association between vegetable fibre intake and depression.
	Fruit fibre	No association between fruit fibre intake and depression.
	Bread/cereal fibre	Negative association between bread/cereal fibre intake and depression.
Kim et al (2020) [S4]	Cereal fibre	No association between cereal fibre intake and clinical depression.
	Vegetable fibre	No association between vegetable fibre intake and clinical depression.
	Fruit fibre	No association between fruit fibre intake and clinical depression.
	Seaweed fibre	Inverse association between cereal fibre intake and clinical depression.
	Mushroom fibre	No association between mushroom fibre intake and clinical depression.
Miki et al (2016) [S5]	SDF	No association between SDF intake and depression.
	IDF	No association between IDF intake and depression.
	Vegetables and fruit fibre	Inverse association between vegetable intake and fruit fibre and depression.
	Cereal fibre	No association between cereal fibre intake and clinical depression.
Xia et al (2021) [S6]	SDF	o No association between SDF intake and depressive symptoms in women<br>o SDF intake associated with lower prevalence of depressive symptoms in men.
	IDF	No association between IDF intake and depressive symptoms in both men and women

	Cereal fibre	No association between cereal fibre intake and depressive symptoms in both men and women
	Vegetable fibre	Vegetable fibre intake associated with lower prevalence of depressive symptoms in both men and women.
	Fruit fibre	No association between fruit fibre intake and depressive symptoms in both men and women
	Soy fibre	Soy fibre intake associated with lower prevalence of depressive symptoms in both men and women.
	Tuber fibre	No association between tuber fibre intake and depressive symptoms in both men and women
Xu et al (2018) [S7]	Cereal fibre	No association between cereal fibre intake and depressive symptoms.
	Vegetable fibre	Inverse association between vegetable fibre intake and depressive symptoms.
	Fruit fibre	Inverse association between fruits fibre intake and depressive symptoms.
Perez-Cornago et al (2016) [S8]	Fructans and GOS	Fructans or GOS intake were not associated with depression
Ramin et al (2020) [S9]	Whole grain fibre and refined fibre	A positive trend between mental health score and whole grain fiber intake and a suggestive inverse trend for refined fiber intake in relation to mental health score were observed.

SDF: Soluble dietary fibre; IDF: Insoluble dietary fibre; FOS: fructooligosaccharides; GOS: galactooligosaccharides

References:

S1. Amadieu C, Leclercq S, Coste V, et al. Dietary fiber deficiency as a component of malnutrition associated with psychological alterations in alcohol use disorder. *Clin Nutr*. 2021;40(5):2673-2682.
S2. Fang CY, Egleston BL, Gabriel KP, et al. Depressive symptoms and serum lipid levels in young adult women. *J Behav Med*. 2013;36(2):143-152
S3. Gopinath B, Flood VM, Burlutksy G, Louie JC, Mitchell P. Association between carbohydrate nutrition and prevalence of depressive symptoms in older adults. *Br J Nutr*. 2016;116(12):2109-2114
S4. Kim C-S, Byeon S, Shin D-M. Sources of dietary fiber are differently associated with prevalence of depression. *Nutrients*. 2020;12(9):2813
S5. Miki T, Eguchi M, Kurotani K, et al. Dietary fiber intake and depressive symptoms in Japanese employees: The Furukawa Nutrition and Health Study. *Nutrition*. 2016;32(5):584-589.
S6. Xia Y, Liu Y, Zhang S, et al. Associations between different types and sources of dietary fibre intake and depressive symptoms in a general population of adults: a cross-sectional study. *Br J Nutr*. 2021;125(11):1281-1290.
S7. Xu H, Li S, Song X, Li Z, Zhang D. Exploration of the association between dietary fiber intake and depressive symptoms in adults. *Nutrition*. 2018;54:48-53.
S8. Perez-Cornago A, Sanchez-Villegas A, Bes-Rastrollo M, et al. Intake of high-fat yogurt, but not of low-fat yogurt or prebiotics, is related to lower risk of depression in women of the SUN cohort study. *The Journal of nutrition*. 2016;146(9):1731-1739.
S9. Ramin S, Mysz MA, Meyer K, Capistrant B, Lazovich D, Prizment A. A prospective analysis of dietary fiber intake and mental health quality of life in the Iowa Women's Health Study. *Maturitas*. 2020;131:1-7.

Additional covariates used for adjusting statistical models in observational studies (extrapolation of Table 1 footnotes)

* Shift work, overtime work, job strain, physical activity, leisure-time physical activity, smoking, alcohol, green tea, coffee, sleep duration, BMI, total energy intake and folate vitamin B6 vitamin B12, n-3 polyunsaturated fatty acids, magnesium, and zinc.

Physical activity, smoking, marital status, socioeconomic status, diabetes, antidepressant use, dietary supplements, dietary intakes of fat, n-3 fatty acids, vitamin B group, total antioxidants, and BMI

$ smoking, drinking, education, household income, employment, marital status, whether one visits friends, household composition, energy intake and family disease history (including CVD, hypertension and diabetes), and dietary pattern scores

^education, income, BMI, energy, smoking, alcohol, physical activity, hypertension, and diabetes

! diabetes, hypertension, hormone replacement therapy, stroke, myocardial infarction, Alzheimer disease, cardiovascular disease, cancer, physical activity, stressful life events, social support, smoking, alcohol, and energy-adjusted intakes of nutrients

Search string

Pubmed

("Dietary Fiber"[Mesh] OR "Dietary Fiber"[tw] OR "Dietary Fibre"[tw] OR “High-fibre*”[tw] OR “High-fiber*”[tw] OR Prebiotic*[tw] OR "Prebiotics"[Mesh] OR Agar[tw] OR Alginate*[tw] OR Carrageenan[tw] OR Cellulose[tw] OR "Cellulose"[Mesh] OR "Pectins"[Mesh] OR Pectin*[tw] OR Pentosan*[tw] OR Polydextrose[tw] OR Polyuronide*[tw] OR Xylose[tw] OR Galactans[tw] OR "Galactans"[Mesh] OR galactooligosaccharide*[tw] OR “galacto-oligosaccharide*”[tw] OR GOS[tw] OR "Fructans"[Mesh] OR Fructan*[tw] OR Inulin[tw] OR Fructooligosaccharide*[tw] OR “Fructo-oligosaccharide*”[tw] OR FOS[tw] OR Oligofructose[tw] OR “Oligo-fructose”[tw] OR Oligosaccharide*[tw] OR “Resistant starch"[Mesh] OR “Resistant starch*”[tw] OR “Psyllium”[Mesh] OR Psyllium[tw] OR ispaghula[tw] OR roughage*[tw]) AND ("Depression"[Mesh] OR Depression[tw] OR "Depressive disorder"[Mesh] OR “Depressive disorder*”[tw] OR “Major depressive disorder*”[tw] OR "Anxiety"[Mesh] OR Anxiety [tw] OR “Anxiety disorder*”[tw] OR“Psychological distress”[tw] OR “Generalised anxiety disorder*”[tw] OR "Mood Disorders"[Mesh] OR Mood[tw]) = 2960

EMBASE

('dietary fiber' OR 'dietary fibre' OR 'dietary fiber'/exp OR 'high-fibre*' OR 'high fiber diet' OR prebiotic* OR 'prebiotic agent'/exp OR 'agar' OR alginate OR carrageenan OR 'cellulose' OR pectin* OR 'pectin'/de OR pentosan* OR polydextrose OR polyuronide* OR xylose OR 'xylose'/de OR galactans OR galactooligosaccharide OR 'galactose oligosaccharide' OR 'galactose' OR gos OR fructans OR inulin OR fructooligosaccharide OR 'fructose oligosaccharide' OR 'fructose oligosaccharide' OR fos OR oligofructose OR 'oligo fructose' OR oligosaccharide* OR 'resistant starch' OR psyllium OR 'ispagula' OR 'roughage') AND ('depression' OR 'depression'/exp OR 'depressive disorder*' OR 'major depressive disorder*' OR 'major depression'/exp OR 'anxiety' OR 'anxiety'/exp OR 'anxiety disorder*' OR 'anxiety

disorder'/exp OR 'psychological distress' OR 'generalised anxiety disorder*' OR 'mood disorder*' OR 'mood disorder'/exp OR mood) AND [humans]/lim AND [embase]/lim = 2160

CINAHL (via EBSCOhost)

("Dietary Fiber" OR "Dietary Fibre" OR “High-fibre*” OR “High-fiber*” OR Prebiotic* OR Agar* OR Alginate* OR Carrageenan OR Cellulose OR Pectin* OR Pentosan* OR Polydextrose* OR Polyuronide* OR Raffinose OR Xylose OR Galactans OR galactooligosaccharide* OR “galacto-oligosaccharide*” OR GOS OR Fructans OR Inulin OR Fructooligosaccharide* OR “Fructo-oligosaccharide*” OR FOS OR Oligofructose OR “Oligo-fructose” OR Oligosaccharide OR “Resistant starch*” OR Psyllium OR Roughage*) AND (Depression OR (MH "Depression+") OR “Depressive disorder*” OR “Major depressive disorder*” OR Anxiety OR (MH "Anxiety+") OR “Anxiety disorder*” OR “Psychological distress” OR “Generalised anxiety disorder*” OR "Mood Disorder*" OR Mood) =553

APA PsycINFO

("Dietary Fiber" OR "Dietary Fibre" OR “High-fibre*” OR “High-fiber*” OR Prebiotic* OR Agar* OR Alginate* OR Carrageenan OR Cellulose OR Pectin* OR Pentosan* OR Polydextrose* OR Polyuronide* OR Raffinose OR Xylose OR Galactans OR galactooligosaccharide* OR “galacto-oligosaccharide*” OR GOS OR Fructans OR Inulin OR Fructooligosaccharide* OR “Fructo-oligosaccharide*” OR FOS OR Oligofructose OR “Oligo-fructose” OR Oligosaccharide OR “Resistant starch*” OR Psyllium OR Roughage*) AND (DE Depression" OR Depression OR "Depressive Disorder" OR “Major depressive disorder*” OR “DE Major depression” OR Anxiety OR DE Anxiety OR “Anxiety disorder*” OR “DE Anxiety disorder*” OR “Psych) =1417

The Cochrane Central Register of Controlled Trials (CENTRAL)

("Dietary Fiber" OR "Dietary Fibre" OR Prebiotic* OR MeSH descriptor: [Prebiotics] explode all trees OR Agar OR MeSH descriptor: [Agar] explode all trees OR Alginate* OR Carrageenan OR Cellulose OR Pectin* OR Pentosan* OR Polydextrose OR Polyuronide* OR Xylose OR Galactans OR MeSH descriptor: [Galactans] explode all trees OR galactooligosaccharide* OR GOS OR Fructans OR Inulin OR Fructooligosaccharide* OR FOS OR Oligofructose OR Oligosaccharide* OR “Resistant starch*” OR Psyllium OR ispaghula OR roughage*) AND (Depression OR MeSH descriptor: [Depression] explode all trees OR "Depressive disorder" OR “Major depressive disorder*” OR Anxiety OR “Anxiety disorder*” OR “Psychological distress” OR “Generalised anxiety disorder*” OR "Mood disorders" OR Mood) =256

Final search conducted on: 20.12.2022
