# Supplementary material for: Fiber intake and fiber intervention in depression and anxiety: a systematic review and meta-analysis of observational studies and randomized controlled trials
Source: Nutr Rev. 2023 Nov 25;82(12):1678–95. doi: 10.1093/nutrit/nuad143 (PMC11551482; doi:10.1093/nutrit/nuad143)
Supplement: nuad143_Supplementary_Data [file nuad143_supplementary_data.zip › nuad143_Supplementary_Data/Appendix_S2.pdf]

Supplemental Table 1. Risk of bias assessment for observational studies

The National Institute of Health (NIH) Quality assessment of Observational Cohort and Cross-Sectional Studies tool

Study		1	2	3	4	5	6	7	8	9	10	11	12	13	14	Overall ROB
Cross-sectional	Amadieu et al (2021)[S1]	Y	Y	Y	Y	Y	N	NA	N	Y	NA	Y	CD	NA	Y	Good
	Chrzastek et al (2020)[S2]	Y	Y	Y	Y	Y	N	NA	N	Y	NA	Y	Y	NA	Y	Good
	Eissenstat et al (2020)[S3]	Y	Y	Y	Y	N	N	NA	N	Y	NA	Y	N	NA	Y	Fair
	Fang et al et al (2013)[S4]	N	Y	Y	Y	N	N	NA	N	Y	NA	Y	Y	NA	Y	Fair
	Gopinath et al (2016) [S5]	Y	Y	Y	Y	N	N	NA	N	Y	NA	Y	NR	NA	Y	Good
	Kim et al (2020) [S6]	Y	Y	N	Y	N	N	NA	N	Y	NA	Y	NR	NA	Y	Fair
	Kim et al (2021) [S7]	Y	Y	N	Y	N	N	NA	N	Y	NA	Y	NR	NA	Y	Fair
	Li et al (2020) [S8]	Y	Y	CD	Y	N	N	NA	Y	Y	NA	Y	CD	NA	Y	Good
	Miki et al (2016) [S9]	Y	Y	Y	Y	N	N	NA	N	Y	NA	Y	CD	NA	Y	Good
	Purnomo et al (2021)[S10]	Y	Y	CD	Y	N	N	NA	N	Y	NA	Y	CD	NA	N	Poor
	Rintamaki et al (2014)[S11]	Y	Y	Y	Y	N	N	NA	N	Y	NA	Y	N	NA	Y	Fair
	Saghafian et al (2021)[S12]	Y	Y	Y	Y	N	N	NA	N	Y	NA	Y	CD	NA	Y	Good
	Woo et al (2006) [S13]	Y	Y	CD	Y	N	N	NA	N	Y	NA	Y	Y	NA	Y	Fair
	Xia et al (2021) [S14]	Y	Y	Y	Y	N	N	NA	N	Y	NA	Y	CD	NA	Y	Good
	Xu et al (2018) [S15]	Y	Y	Y	Y	N	N	NA	N	Y	NA	Y	CD	NA	Y	Good
	Yun et al (2021) [S16]	Y	Y	Y	Y	N	N	NA	N	N	NA	Y	CD	NA	Y	Poor
Longitudinal	Gangwisch et al (2015)[S17]	Y	Y	NR	Y	N	Y	Y	N	Y	NR	Y	CD	CD	Y	Fair
	Perez-Cornago et al (2016) [S18]	Y	Y	Y	Y	NR	Y	Y	N	Y	Y	Y	CD	Y	Y	Good
	Ramin et al (2020) [S19]	Y	Y	Y	Y	NR	Y	CD	N	Y	NR	Y	CD	Y	Y	Fair

The National Institute of Health (NIH) Quality assessment of assessment of Case Control Studies tool

Case-control		1	2	3	4	5	6	7	8	9	10	11	12	Overall ROB
	Gougeon et al (2017) [S20]	Y	Y	N	Y	Y	Y	NR	Y	Y	Y	CD	Y	Good
	Guligowska et al (2016) [S21]	Y	Y	N	Y	Y	Y	NR	Y	N	Y	CD	N	Fair

Othman et al (2018) [S22]	Y	N	N	NR	N	N	NR	Y	NA	N	CD	N	Poor
Park et al (2010) [S23]	Y	Y	N	Y	N	Y	NR	CD	NA	N	CD	N	Poor

*CD: cannot determine; NA: not applicable; NR: not reported

Key: NIH Quality assessment of Observational Cohort and Cross-Sectional Studies tool

1. Was the research question or objective in this paper clearly stated?
2. Was the study population clearly specified and defined?
3. Was the participation rate of eligible persons at least 50%?
4. Were all the subjects selected or recruited from the same or similar populations (including the same time period)? Were inclusion and exclusion criteria for being in the study prespecified and applied uniformly to all participants?
5. Was a sample size justification, power description, or variance and effect estimates provided?
6. For the analyses in this paper, were the exposure(s) of interest measured prior to the outcome(s) being measured?
7. Was the timeframe sufficient so that one could reasonably expect to see an association between exposure and outcome if it existed?
8. For exposures that can vary in amount or level, did the study examine different levels of the exposure as related to the outcome (e.g., categories of exposure, or exposure measured as continuous variable)?
9. Were the exposure measures (independent variables) clearly defined, valid, reliable, and implemented consistently across all study participants?
10. Was the exposure(s) assessed more than once over time?
11. Were the outcome measures (dependent variables) clearly defined, valid, reliable, and implemented consistently across all study participants?
12. Were the outcome assessors blinded to the exposure status of participants?
13. Was loss to follow-up after baseline 20% or less?
14. Were key potential confounding variables measured and adjusted statistically for their impact on the relationship between exposure(s) and outcome(s)?

Key: NIH Quality assessment of Case Control Studies tool

1. Was the research question or objective in this paper clearly stated and appropriate?
2. Was the study population clearly specified and defined?
3. Did the authors include a sample size justification?
4. Were controls selected or recruited from the same or similar population that gave rise to the cases (including the same timeframe)?

5. Were the definitions, inclusion and exclusion criteria, algorithms or processes used to identify or select cases and controls valid, reliable, and implemented consistently across all study participants?

6. Were the cases clearly defined and differentiated from controls?

7. If less than 100 percent of eligible cases and/or controls were selected for the study, were the cases and/or controls randomly selected from those eligible?

8. Was there use of concurrent controls?

9. Were the investigators able to confirm that the exposure/risk occurred prior to the development of the condition or event that defined a participant as a case?

10. Were the measures of exposure/risk clearly defined, valid, reliable, and implemented consistently (including the same time period) across all study participants?

11. Were the assessors of exposure/risk blinded to the case or control status of participants?

12. Were key potential confounding variables measured and adjusted statistically in the analyses? If matching was used, did the investigators account for matching during study analysis?

References:

S1. Amadieu C, Leclercq S, Coste V, et al. Dietary fiber deficiency as a component of malnutrition associated with psychological alterations in alcohol use disorder. *Clin Nutr*. 2021;40(5):2673-2682.
S2. Chrzastek Z, Guligowska A, Piglowska M, Soltysik B, Kostka T. Association between sucrose and fiber intake and symptoms of depression in older people. *Nutr Neurosci*. 2022;25(5):886-897.
S3. Eissenstat SJ, Gao N, Radler D, Oh TL. Nutrient intake differences among ethnic groups and risks of depression. *Journal of Immigrant and Minority Health*. 2020;22(6):1141-1148.
S4. Fang CY, Egleston BL, Gabriel KP, et al. Depressive symptoms and serum lipid levels in young adult women. *J Behav Med*. 2013;36(2):143-152
S5. Gopinath B, Flood VM, Burlutksy G, Louie JC, Mitchell P. Association between carbohydrate nutrition and prevalence of depressive symptoms in older adults. *Br J Nutr*. 2016;116(12):2109-2114
S6. Kim C-S, Byeon S, Shin D-M. Sources of dietary fiber are differently associated with prevalence of depression. *Nutrients*. 2020;12(9):2813
S7. Kim Y, Hong M, Kim S, Shin W-y, Kim J-h. Inverse association between dietary fiber intake and depression in premenopausal women: a nationwide population-based survey. *Menopause*. 2021;28(2):150-156.
S8. Li D, Tong Y, Li Y. Dietary fiber is inversely associated with depressive symptoms in premenopausal women. *Front Neurosci*. 2020;14:373.
S9. Miki T, Eguchi M, Kurotani K, et al. Dietary fiber intake and depressive symptoms in Japanese employees: The Furukawa Nutrition and Health Study. *Nutrition*. 2016;32(5):584-589.
S10. Purnomo J, Jeganathan S, Begley K, Houtzager L. Depression and dietary intake in a cohort of HIV-positive clients in Sydney. *Int J STD AIDS*. 2012;23(12):882-886.
S11. Rintamäki R, Kaplas N, Männistö S, et al. Difference in diet between a general population national representative sample and individuals with alcohol use disorders, but not individuals with depressive or anxiety disorders. *Nordic journal of psychiatry*. 2014;68(6):391-400
S12. Saghafian F, Sharif N, Saneei P, et al. Consumption of dietary fiber in relation to psychological disorders in adults. *Frontiers in Psychiatry*. 2021:926.
S13. Woo J, Lynn H, Lau W, et al. Nutrient intake and psychological health in an elderly Chinese population. *International Journal of Geriatric Psychiatry: A journal of the psychiatry of late life and allied sciences*. 2006;21(11):1036-1043.
S14. Xia Y, Liu Y, Zhang S, et al. Associations between different types and sources of dietary fibre intake and depressive symptoms in a general population of adults: a cross-sectional study. *Br J Nutr*. 2021;125(11):1281-1290.
S15. Xu H, Li S, Song X, Li Z, Zhang D. Exploration of the association between dietary fiber intake and depressive symptoms in adults. *Nutrition*. 2018;54:48-53.
S16. Yun H, Kim D-W, Lee E-J, Jung J, Yoo S. Analysis of the effects of nutrient intake and dietary habits on depression in Korean adults. *Nutrients*. 2021;13(4):1360.
S17. Gangwisch JE, Hale L, Garcia L, et al. High glycemic index diet as a risk factor for depression: analyses from the Women's Health Initiative. *The American journal of clinical nutrition*. 2015;102(2):454-463.
S18. Perez-Cornago A, Sanchez-Villegas A, Bes-Rastrollo M, et al. Intake of high-fat yogurt, but not of low-fat yogurt or prebiotics, is related to lower risk of depression in women of the SUN cohort study. *The Journal of nutrition*. 2016;146(9):1731-1739.
S19. Ramin S, Mysz MA, Meyer K, Capistrant B, Lazovich D, Prizment A. A prospective analysis of dietary fiber intake and mental health quality of life in the Iowa Women's Health Study. *Maturitas*. 2020;131:1-7.
S20. Gougeon L, Payette H, Morais JA, Gaudreau P, Shatenstein B, Gray-Donald K. A prospective evaluation of the depression–nutrient intake reverse causality hypothesis in a cohort of community-dwelling older Canadians. *Br J Nutr*. 2017;117(7):1032-1041.
S21. Guligowska A, Pigłowska M, Fife E, et al. Inappropriate nutrients intake is associated with lower functional status and inferior quality of life in older adults with depression. *Clin Interv Aging*. 2016;11:1505.
S22. Ben Othman R, Mziou O, Gamoudi A, et al. Nutritional Status of Depressive Patients. *J Diabetes Metab*. 2018;9(2)
S23. Park J-Y, You J-S, Chang K-J. Dietary taurine intake, nutrients intake, dietary habits and life stress by depression in Korean female college students: a case-control study.*J Biomed Sci*. 2010;17(1):1-5.

Supplemental Table 2. Risk of bias assessment for randomised controlled trials

Cochrane-risk-of bias tool						
Study	Domain 1: Risk of bias arising from the randomization process	Domain 2: Risk of bias due to deviations from the intended interventions 2	Domain 3: Risk of bias due to missing outcome data	Domain 4: Risk of bias in measurement of the outcome	Domain 5: Risk of bias in selection of the reported result 5	Overall ROB
Azpiroz et al (2017) [S1]	Low risk	Some concerns	Low risk	Low risk	Low risk	Some concerns
Farhangi et al (2018) [S2]	Low risk	Low risk	Low risk	Low risk	Low risk	Low risk
Ibarra et al (2016) [S3]	Low risk	Low risk	Low risk	Low risk	Low risk	Low risk
Johnstone et al (2021) [S4]	Low risk	Low risk	Low risk	Low risk	Low risk	Low risk
Kazemi et al (2019) [S5]	Low risk	Low risk	Low risk	Low risk	Low risk	Low risk
Moludi et al (2021) [S6]	Low risk	Low risk	Low risk	Low risk	Low risk	Low risk
Schmidt et al (2014) [S7]	Low risk	Low risk	Low risk	Low risk	Some concern	Some concern
Silk et al (2009) [S8]	Low risk	Low risk	Low risk	Some concern	Low risk	Some concern
Vulevic et al (2018) [S9]	Low risk	Low risk	Low risk	Low risk	Some concern	Some concern
Smith et al (2005) [S10]	High risk	Some concerns	Low risk	High risk	Some concern	High risk

References:

S1. Azpiroz F, Dubray C, Bernalier-Donadille A, et al. Effects of sc FOS on the composition of fecal microbiota and anxiety in patients with irritable bowel syndrome: A randomized, double blind, placebo controlled study. *Neurogastroenterol Motil*. 2017;29(2):e12911.

S2. Farhangi MA, Javid AZ, Sarmadi B, Karimi P, Dehghan P. A randomized controlled trial on the efficacy of resistant dextrin, as functional food, in women with type 2 diabetes: Targeting the hypothalamic–pituitary–adrenal axis and immune system. *Clin Nutr*. 2018;37(4):1216-1223.

S3. Ibarra A, Olli K, Pasman W, et al. Effects of polydextrose with breakfast or with a midmorning preload on food intake and other appetite-related parameters in healthy normal-weight and overweight females: An acute, randomized, double-blind, placebo-controlled, and crossover study. *Appetite*. 2017;110:15-24.

S4. Johnstone N, Milesi C, Burn O, et al. Anxiolytic effects of a galacto-oligosaccharides prebiotic in healthy females (18–25 years) with corresponding changes in gut bacterial composition. *Sci Rep*. 2021;11(1):1-11.

S5. Kazemi A, Noorbala AA, Azam K, Eskandari MH, Djafarian K. Effect of probiotic and prebiotic vs placebo on psychological outcomes in patients with major depressive disorder: A randomized clinical trial. *Clin Nutr*. 2019;38(2):522-528.

S6. Moludi J, Khedmatgozar H, Nachvak SM, Abdollahzad H, Moradinazar M, Sadeghpour Tabaei A. The effects of co-administration of probiotics and prebiotics on chronic inflammation, and depression symptoms in patients with coronary artery diseases: a randomized clinical trial. *Nutr Neurosci*. 2022;25(8):1659-1668.

S7. Schmidt K, Cowen PJ, Harmer CJ, Tzortzis G, Errington S, Burnet PW. Prebiotic intake reduces the waking cortisol response and alters emotional bias in healthy volunteers. *Psychopharmacology*. 2015;232(10):1793-1801.

S8. Silk D, Davis A, Vulevic J, Tzortzis G, Gibson G. Clinical trial: the effects of a trans-galactooligosaccharide prebiotic on faecal microbiota and symptoms in irritable bowel syndrome. *Aliment Pharmacol Ther*. 2009;29(5):508-518.

S9. Vulevic J, Tzortzis G, Juric A, Gibson GR. Effect of a prebiotic galactooligosaccharide mixture (B-GOS®) on gastrointestinal symptoms in adults selected from a general population who suffer with bloating, abdominal pain, or flatulence. *Neurogastroenterol Motil*. 2018;30(11):e13440.

S10. Smith AP. The concept of well-being: relevance to nutrition research. *Br J Nutr*. 2005;93(S1):S1-S5.
